# A comparative study of patients’ activities and interactions in a stroke unit before and after reconstruction—The significance of the built environment

**DOI:** 10.1371/journal.pone.0177477

**Published:** 2017-07-20

**Authors:** Anna Anåker, Lena von Koch, Christina Sjöstrand, Julie Bernhardt, Marie Elf

**Affiliations:** 1 Karolinska Institutet, Department of Neurobiology, Care Sciences and Society, Stockholm, Sweden; 2 Dalarna University, School of Education, Health and Social Studies, Falun, Sweden; 3 Karolinska University Hospital, Department of Neurology, Stockholm, Sweden; 4 Karolinska Institutet, Department of Clinical Neuroscience, Stockholm, Sweden; 5 Florey Institute of Neuroscience and Mental Health, Melbourne, Victoria, Australia; 6 Chalmers University of Technology, School of Architecture, Gothenburg, Sweden; IRCCS E. Medea, ITALY

## Abstract

Early mobilization and rehabilitation, multidisciplinary stroke expertise and comprehensive therapies are fundamental in a stroke unit. To achieve effective and safe stroke care, the physical environment in modern stroke units should facilitate the delivery of evidence-based care. Therefore, the purpose of this study was to explore patients’ activities and interactions in a stroke unit before the reconstruction of the physical environment, while in a temporary location and after reconstruction. This case study examined a stroke unit as an integrated whole. The data were collected using a behavioral mapping technique at three different time points: in the original unit, in the temporary unit and in the new unit. A total of 59 patients were included. The analysis included field notes from observations of the physical environment and examples from planning and design documents. The findings indicated that in the new unit, the patients spent more time in their rooms, were less active, and had fewer interactions with staff and family than the patients in the original unit. The reconstruction involved a change from a primarily multi-bed room design to single-room accommodations. In the new unit, the patients’ lounge was located in a far corner of the unit with a smaller entrance than the patients’ lounge in the old unit, which was located at the end of a corridor with a noticeable entrance. Changes in the design of the stroke unit may have influenced the patients’ activities and interactions. This study raises the question of how the physical environment should be designed in the future to facilitate the delivery of health care and improve outcomes for stroke patients. This research is based on a case study, and although the results should be interpreted with caution, we strongly recommend that environmental considerations be included in future stroke guidelines.

## Introduction

People who suffer from a stroke often face challenges in recovering their ability to perform every-day tasks, including communicating through spoken and written language, walking or managing fatigue [1, 2]. Evidence confirms that treatment and care at dedicated stroke units can contribute to the overall health and well-being of people recovering from stroke [3]. The vast majority of Swedish stroke patients are cared for in stroke units [4]. Rehabilitation at stroke units results in more patients surviving, returning home, and regaining independence in daily activities compared with rehabilitation in general wards [5]. Stroke units ensure the co-location of people affected by stroke, who are treated in a geographically bounded area by multidisciplinary staff with specific expertise in stroke care [3]. However, the contribution of the physical environment to effective stroke care is poorly understood.

Current guidelines for stroke care recommend starting rehabilitation early to regain functions, such as the ability to speak and walk, and to reduce complications [3, 5]. Observations of patients’ activities and interactions in stroke units have revealed that patients are often alone and inactive during the acute phase [6–9]. Similar inactivity patterns were found in a recent study of eleven Norwegian stroke units, but an increase in upright patient activity was observed in hospitals where meals were served in a communal area [10]. This emphasizes the need to further elucidate whether and how the physical environment can be a barrier to or a facilitator of patients’ activities and interactions in stroke units.

Today, there is growing evidence that the design of the physical environment can affect patients’ health, care and well-being [11, 12]. Research on the physical health care environment emphasizes the connection between the design of the physical environment and a range of health outcomes [11–14]. For example, patients with access to natural light have been found to have a shorter length of stay and to use less analgesic medication [12]. Furthermore, poor acoustic surroundings, e.g., noise from technical machinery, have been associated with higher rehospitalization rates [15].

In the International Classification of Functioning, Disability and Health (ICF) framework, The World Health Organization (WHO) highlights the importance of the environment to an individuals’ functioning [16]. According to the ICF, an individual’s health is the result of the interaction between the individual’s functioning and contextual factors, such as the design of the person’s environment, which can facilitate or create barriers to the individual's activity and participation [17].

The evidence-based design (EBD) model has been developed over the past decade and advocates that fundamental decisions regarding the built environment should be based on the best available knowledge from research and practice [18, 19]. Studies have demonstrated an interdependency between the physical environment, patient care and rehabilitation after stroke that may affect a patients’ recovery after stroke, in some cases hindering the recovery process [20, 21]. Thus, it is of utmost importance to increase our understanding of whether and how the physical environment can support an individual’s ability to adapt to and interact with his or her immediate surroundings after stroke.

How the physical environment itself might facilitate or hinder evidence-based and safe stroke care needs further exploration. In this study, we aimed to measure and explore patients’ activities and interactions in a stroke unit before the reconstruction of the physical environment, while in a temporary location and after reconstruction. A further aim was to relate activities and interactions with changes in the design features across these three phases of reconstruction.

## Materials and methods

### Design

The study was an explorative case study [22] that examined a stroke unit as an integrated whole.

### Setting and participants

The study was conducted in a stroke unit at a Swedish university hospital that was undergoing rebuilding. There were two stages in the rebuilding process: the relocation of the original staff to temporary accommodations while the old unit was demolished and the rebuilding of a new unit on the old site. These stages resulted in the three distinct ‘stroke unit’ conditions examined in this study: the original unit, the temporary unit and the new unit.

Patients who met the inclusion criteria were recruited consecutively. Patients were eligible for inclusion if they a) had a confirmed stroke diagnosis, b) had been admitted to the stroke unit for at least 24 hours, and c) could answer questions. A total of 59 patients were included in the study: 22 were observed in the original unit, 21 in the temporary unit and 16 in the new unit. All the included patients were able to perform activities (e.g., stand, walk, eat, sit in bed, sit out of bed) the day of the observation. The unit included both patients in the acute phase of stroke who required medical care and monitoring and patients who were mainly receiving rehabilitation services. Patients who were receiving palliative care were excluded.

### Data collection

Data collection was performed in the original unit for two weeks in April 2013, in the temporary unit for two weeks in October 2013 and finally in the new stroke unit for two weeks in December 2015. At the time the observations in the new unit were conducted, the staff had been working there for thirteen months.

The data were derived via a behavioral mapping technique, which is a standardized and frequently used method for quantifying the amount and nature of patients’ activity, their location in a setting and the other people present [6]. The method has exhibited good validity [23] and good interobserver reliability [6]. Data collection was performed by three trained observers. The participants (staff and patients) were informed about the study's purpose and asked to avoid performing any unusual work during the observation day. Each participant was observed over one weekday from 8 am to 5 pm. Observations were recorded every 10 minutes on a pre-defined route in the stroke unit that remained consistent throughout the day. At each observation point, the category of activity (e.g., talking, eating, sitting supported out of bed, walking or standing), the people present during the activity/interactions (e.g., none (alone), nurse, physician, therapist or significant other) and the location of the activity (e.g., patient’s room, corridor, therapy area or patient lounge) were recorded. Given the design of the physical environment, field notes based on Spradly’s [24] nine dimensions of social situations (e.g., physical place, the people involved, activities and the physical things present) were included in the results. Furthermore, examples from the planning and design documents (retrieved from the Building and Planning Department) were included and used in the results to provide a rich description of the physical environment.

### Data processing analysis

Once completed, the behavioral mapping forms were scanned as PDF documents and sent to the central processing office at the Florey Institute, Melbourne, Australia. The quantifiable data analysis of the descriptive statistics was conducted using IBM SPSS 23. The results are expressed in relative numbers as a location over time, with 100% referring to a location that lasted a full day (from 8 am to 5 pm). The activities were categorized based on their level of therapeutic value [6] (Table 1).

**Table 1:** Observed activities organized into categories.

Activity category	Activity
No activity	No motor activity
Minimal activity	Talking, reading, eating, using arms, sitting supported in bed
Low activity	Sitting supported out of bed, sitting in hoist, transferring
Moderate activity	Rolling and sitting up, sitting unsupported, transferring feet onto floor
High activity	Standing, walking, using stairs

### Ethical permission

The study was approved by a local Ethics Committee of Sweden (permit numbers: EPN No. 2012/199). Written and oral informed consent was obtained from all the participants prior to data collection. The participants were free to withdraw from the study at any time.

## Results

A total of 3,116 observations were performed on a total of 59 patients. The patients’ characteristics are presented in Table 2.

**Table 2:** Characteristics of the included patients.

Variable	Original unit	n (%)	Temporary unit	n (%)	New unit	n (%)
**N**	22		21		16	
**Age, mean (SD)**	75.8 (14.0)		78.3 (15.0)		75.9 (12.6)	
**Sex Female**		11 (50.0)		11 (52.4)		5 (31.2)
**First Stroke**		15 (68.2)		19 (90.5)		12 (75.0)
**Time since stroke in days, median (IQR**^a^**)**	13.0 (27.5)		4.0 (5.0)		9.5 (20.5)	
**Infarct**		16 (72.7)		17 (80.9)		15 (93.7)
**Haemorrhage**		3 (13.6)		3 (14.3)		1 (6.3)
**Missing**		3 (13.6)		1 (4.8)		0 (0.0)
**NIHSS**^b^ **median (IQR)**	2.0 (5.8)		3.5 (5.8)		4.5 (9.5)	
**Mild (0–7)**	2.0 (5.0)	14 (63.6)	3.0 (4.1)	17 (80.9)	3.0 (3.0)	11 68.7)
**Moderate (8–16)**	14.0 (11–16*)	3 (13.6)	9.5 (9–10*)	2 (9.5)	13.0 (4.5)	5 (31.3)
**Severe (>16)**	17.0 (17.0)	1 (4.5)	24.0 (24.0)	1 (4.8)	0 (0.0)	0 (0.0)
**Missing**		4 (18.2)		1 (4.8)		0 (0.0)

^a^ IQR, Interquartile Range

^b^ NIHSS, National Institutes of Health Stroke Scale. Day of arrival in the unit.

* Range

### Characteristics of the physical environment of the stroke units

The original stroke unit was built in 1974 and had not been restored since. In 2014, a new, renovated stroke unit was launched. According to the design and building documents, the rebuilt and modernized stroke unit was intended to be safe and to focus on infection control. To increase flexibility and facilitate the future transition of the unit, all the floors of the building were renovated in a standard manner, i.e., all the floors were identical.

The original and temporary units were based mainly on a multi-bed room design, whereas the new unit primarily has single rooms. The intention of designing the new unit with mainly single rooms was to enhance privacy and decrease the risk of infection by housing each patient in a single room with a private shower and toilet. According to the planning and design documents, the patient rooms, corridors and therapy areas in the new unit should be perceived as safe and pleasant. The new unit was also designed to incorporate direct daylight and to have windows that provided views from the rooms (Table 3).

**Table 3:** Characteristics of the physical environment of the stroke unit during different rebuilding phases (based on field notes and planning and design documents).

Location	Environmental characteristic	Original unit	Temporary unit	New unit
**Stroke unit**	General characteristics of the stroke unit	Two parallel corridors with two nursing stations, one on each side. Separate rooms for physicians and other health professionals.	Two parallel corridors with one nursing station. Separate rooms for physicians and other health professionals.	Two parallel corridors with four nursing stations (so-called team stations), two on each side. Separate rooms for physicians and other health professionals.
**Patient’s room**	Multi-bed rooms (2–4 patients/room)	Yes	Yes	No (One room reserved for acute patients (n = 3) in need of medical monitoring)
	Single rooms	No (The unit had three single rooms for patients with special needs, e.g., infection control)	No	Yes
	Windows	Windows allowing view from the room	Windows allowing view from the room	Windows allowing view from the room
	Light source	Daylight + artificial	Daylight + artificial	Daylight + artificial
	Sound from staff, other patients and significant others	High	High	Low
	Doors to the corridor remained open throughout the day of observation	Yes, the doors were often opened throughout the day	Yes, the doors were often opened throughout the day	No, the doors were often closed throughout the day
	Doors with windows	No	No	Yes
**Bathroom with toilet**	Location of the bathroom and toilet	Outside the room	Outside the room	In the patient room
	Light source	Artificial	Artificial	Artificial (motion detector)
**Corridor**	Obstacles, e.g., medication carts and wheelchairs along the walls	Yes	Yes	Yes
	Window allowing view from the corridor	No	No	No
	Light source	Artificial	Artificial	Artificial
	Handrails along the walls	Yes	Yes	Yes
**Therapy area**	Window with view	Yes	Yes	Yes
	Light source	Daylight + artificial	Daylight + artificial	Daylight + artificial
**Patient lounge**	Placed at the end of one corridor	Yes	No, the lounge was located in a former patient bedroom. Only six patients were allowed to visit the room at the same time.	Yes
	Noticeable entrance	Yes, with a large open entrance at one end of the corridor	No, it was not possible to see the entrance when standing in the corridor	No, it was not possible to see the entrance when standing in the corridor

### Where patients spent their day

An analysis of the proportion of the day that patients spent in different locations in the stroke units showed that the patients in the original unit were in their room half of the day. When studying the location pattern in the temporary unit, the time patients spent in their rooms increased. In the new stroke unit, the patients were in their room most of the time. Only small portions of the day were spent in areas outside the patients’ bedrooms (Table 4).

**Table 4:** Proportion (%) of the day spent in different locations in the stroke unit.

	*Proportion of the day (%)*		
*Location*	Original unit	Temporary unit	New unit
**Bathroom**	3.5	2.5	2.3
**Patient’s room**	54.8	76.3	83.1
**Corridor**	9.3	7.5	3.4
**Therapy area**	5.2	0.7	0.6
**Patient lounge**	12.2	3.2	8.6
**Physicians room**	0.2	0	0.6
**Off ward**	2.1	3.6	1.2
**Other**	0.5	0.9	0.0
**Missing**	12.2	5.3	0.2

### Time with people—Interactions

The patients in the original unit were alone for half of the observation day (Table 5). In the temporary unit, the proportion of the day the patients were alone was larger, and in the new unit, the proportion was even larger. In all the observed units, the most frequent contact the patients had in their patient rooms was with significant others. The people present were not mutually exclusive, and the proportions in the table did not sum to 100% because the patients were occasionally observed to interact with more than one person on the same occasion.

**Table 5:** Proportion (%) of the day spent alone or with people present.

	*Proportion of the day (%)*		
*People present*	Original unit	Temporary unit	New unit
**Alone**	49.6	63.6	82.8
**Physicians**	1.3	1.6	0.4
**Nurses**	3.2	3.4	2.4
**Nurse assistants**	7.4	7.6	5.3
**Physiotherapist**	4.9	2.1	2.2
**Occupational therapist**	2.9	2.2	1.2
**Speech & language therapist**	2.6	0.7	0.2
**Significant others**	10.6	8.2	6.3
**Another team member**	0.2	1.0	0.6
**Interpreter**	1.9	0.6	0
**Other (e.g., priest, librarian)**	5.8	4.5	0.0
**≥ Two staff and/or family members at the same time**	4.8	4.8	1.9

### Activities

The results of the observations of the patients’ activities are displayed in Table 6. The proportion of time spent in each activity category indicated that the time spent engaged in no activities increased when comparing the new unit with the original unit. The proportion of the day that the patients were engaged in activities in the high-activity category was lower in the new unit than in the original unit.

**Table 6:** Patients’ activities as a proportion (%) of the day spent in different activity categories.

	*Proportion of the day (%)*					
*Activity level*	No activity	Minimal activity	Low activity	Moderate activity	High activity	Missing
**Original unit**	25.3	9.1	21.7	22.0	7.0	14.9
**Temporary unit**	39.5	14.4	9.5	21.6	5.0	10.0
**New unit**	54.1	8.1	30.9	0.5	4.0	2.4

## Discussion

In this case study, we had the unique opportunity to explore and compare patients’ activities and interactions in three distinct physical stroke unit environments: an old unit, a temporary unit and a newly renovated unit. The findings indicated that patients’ activities and interactions varied among the units and according to their design. In the new stroke unit, the patients spent more time alone in their rooms, were less active, and had fewer interactions than the patients in the original unit.

The findings in our study are of clinical importance as the design of the physical environment of the new stroke unit did not appear to support the recommendations in the current guidelines for stroke care, e.g., early rehabilitation and continuous observation of patients’ health status [25]. Research has shown that early rehabilitation after stroke could promote better health outcomes [26] and that the rehabilitation process is likely to be promoted if the patient is engaged in physical activities or social interactions [3, 27]. Instead, in the new stroke unit, the patients spent a larger proportion of the day doing nothing than the patients in the old stroke unit did. That people with stroke spent most of their time inactive and alone was consistent with activity patterns reported in several previous studies [7, 28, 29]. To improve patients’ activity levels, the design and construction of a new unit should include discussions of how the environment will promote physical activity and social interactions with staff and others.

During the rebuilding phase, some structural and design changes were implemented. For example, the unit was modified from a mainly multi-bed room structure to single-room accommodations, which may in part explain why patients remained in their rooms and were less active. One plausible explanation is that the single-room design of the new unit led to more isolation and inactivity among the patients. This interpretation is consistent with results from other studies that have found that single-room designs were associated with fewer interactions with other patients and staff [30, 31].

There is an ongoing debate regarding whether hospital units should be designed exclusively with single rooms or multi-bed rooms, with a general trend towards the provision of single rooms in new health care buildings [30, 32, 33]. The most important argument for a single-room design is the reduction of airborne- and contact-transmitted infections [12]. Single rooms are also associated with reductions in noise [12], the number of harmful and costly patient transfers [34, 35], and improved communication between staff and patients because of enhanced patient privacy [12, 36, 37]. All these advantages are undoubtedly important for patients with stroke; however, when early rehabilitation is a central aspect of the care in stroke units, the design of the environment should also support and encourage activity and interaction.

In recent years, several studies have highlighted the complexity of the issues related to single rooms [30, 32, 38]. While some patients have reported that they preferred single rooms because the rooms allowed them to create a personal and private environment [32], staff have reported disadvantages related to visibility, surveillance, teamwork, monitoring and keeping patients safe [30]. A recent study [32] of patients’ experiences of being cared for in single rooms concluded that it is not natural for patients to move around outside their bed areas when they have all they need in their rooms. Furthermore, the patients reported that single rooms allowed them to create a personal and private environment but that such rooms also generated a feeling of loneliness. Additionally, in our study, it appeared that patients preferred to remain in their rooms once they were admitted to a single room.

The results of our study shed light on the importance of matching the design of the environment to the organization of care. An old organizational model with a multi-bed room layout in which a nurse can observe multiple patients simultaneously combined with a lack of staff might jeopardize the good intentions of such a design. An analysis of care processes and organization must be conducted in parallel with planning new environments. In addition, the effects of introducing exclusively single rooms in new hospital buildings and how to counteract the disadvantages of single rooms need to be further investigated. We observed that when patients were admitted to single rooms in the new unit, the doors to the corridor remained closed all day, which may have made the patients less likely to go out into the corridor and therefore less likely to take a walk in the corridor. The patients also spent less time in the therapy area than they did in the old unit, which may suggest that therapy took place more often in patients’ single rooms.

Furthermore, some stroke patients could have cognitive impairments that might result in difficulties with navigating the unit. In the new unit, the patients’ lounge was located in a far corner of the stroke unit with a smaller entrance than the patients’ lounge in the old stroke unit, which was located at the end of a corridor with a noticeable entrance. The lack of a clear and easily navigated physical environment may have contributed to the patients’ reluctance to leave their room. According to previous research [12, 33], successful spatial navigation is an important element of hospital ward design, and inadequate navigation might contribute to a sense of lost control over the situation [33]. Had the patient lounge been placed more centrally in the ward, it might have increased access and the time patients spent in the communal areas. Access to meeting places, such as patient lounges, and opportunities for cognitive and social activities, e.g., access to computers, books, newspapers, games and personal hobbies, have been reported to promote activity and well-being among people with stroke [10, 20, 39]. We argue that a clearly visible and inviting communal area and a simple and understandable corridor plan offering views from windows to support orientation could facilitate and encourage patients to be more physically active and engage in more extensive interactions with other people.

The new stroke unit in our study was built in accordance with a standard model: all the units and wards in the hospital were designed using the same predetermined criteria. Different patient groups may have different needs and requirements from their physical environments. For example, a patient who is infected should be in a room with a closed door for infection control, but patients in rehabilitation who should be active require rooms that encourage activity in areas that stimulate interaction.

People live their lives as part of the built environment [40], and according to the ICF, environmental factors “make up the physical, social and attitudinal environment in which people live and conduct their lives” [17]. Environmental factors are not specifically mentioned in the current stroke guidelines. We strongly suggest that stroke guidelines consider the physical environment and outline special features of the environment that are intended to support patients’ health and recovery after a stroke. Based on our findings, we conclude that it is time to consider the environment in which care is delivered as well as the care itself. However, more knowledge is needed before design recommendations can be made. Non-supportive designed environments may increase the amount of time patients spend alone and unengaged in activities and interactions that might promote the rehabilitation process.

The design of modern stroke units should be based on evidence-based design solutions and information about the patient group that will occupy the environment. According to an evidence-based design [41], the environment should be functional, safe and stimulating. Our findings underpin the recommendation that the planning and design of new health care environments should consider the demands of the care organization, care processes and patients’ needs [42]. These discussions should also consider questions regarding how to account for environmental factors in the design of new stroke units. Further quantitative and qualitative research is needed to extend the findings of this explorative study of the impact of the physical environment on patient care.

### Strengths and limitations

The strength of this study is that we had a unique opportunity to observe a stroke unit over time through all the phases of its rebuilding process. Another strength is our use of behavioral mapping, which is a standardized method used to quantify the amount and nature of patients’ activity, their location in a setting and the other people present. However, there are some limitations that should be considered. There are some data missing from the data collection; however, the same trends were observed in the results regardless of the missing data. Observational studies have the potential for bias due to the observers’ influence on the research participants’ behavior. There is the possibility that we overestimated the degree of physical activity since the participating patients may have been more active when we observed them. However, research has indicated [43] that after a few minutes of observation, the participants in observational studies return to their normal behaviors, and the observer becomes a subordinate person. The present study included a limited number of patients, and caution should be exerted regarding generalization to other Swedish or international units. A further limitation was the exclusion of patients receiving palliative care, which means that the suitability of the physical environment for this group was not considered. This study included 9 hours of observation for each patient; thus, future research including evening observations is of interest.

## Conclusion

This study highlighted that patients in the new stroke unit spent more time in their rooms, were less active, and had fewer interactions than patients in the original unit. Furthermore, this study raises the question of how physical environments should be designed in the future to best meet health care requirements regarding early mobilization and rehabilitation with the ultimate goal of providing quality care for all people. We strongly recommend that environmental factors be included in future stroke guidelines. Information about the factors that influence the care of patients treated in a stroke unit must be integrated into future planning and design processes for physical environments, with the ultimate goal of producing an evidence-based design.

## Supporting information

S1 Fig Stroke 1. (PDF)

S2 Fig Stroke 2. (PDF)

S3 Fig Stroke 3. (PDF)
