# Supplementary material for: A comparative study of patients’ activities and interactions in a stroke unit before and after reconstruction—The significance of the built environment
Source: PLoS One. 2017 Jul 20;12(7):e0177477. doi: 10.1371/journal.pone.0177477 (PMC5519004; doi:10.1371/journal.pone.0177477)
Supplement: S1 Fig — (PDF) [file pone.0177477.s001.pdf]

TIME	Location	People	Activity
800	2	1	1
810	2	1	5,6
820	2	1	5,6
830	2	1	1
840	2	14	4
850	2	14	4
900	2	14	6
910	1		
920	1	1	12,13
930	1	2	13
940	2	2,3,14	4,10
950	2	1	10
1000	1	1	12,13
1010	2	1	4,15,16
1020	2	1	4,10
1040	2	1	4,10
1050	2	1	13
1100	2	1	12,13
1110	2	14	4,10
1120	2	14	4,10
1130	2	14	10
1140	2	14	4,10
1150	2	14	4,10
1200	2	14	4,10
1210	2	14	4,10
1220	3	3,14	4,10
1230	3	3,14	4,10
1240	3	14	4,10
1250	3	14	4,10
1300	3	14	4,10
1310	2	1	5,10
1320	2	1	5,10
1340			17
1350			17
1400			17
1410			17
1420			17
1430			17
1440			17
1450			17
1500			17
1510			17
1520			17
1530			17
1540			17
1550			17
1600			17
1620			17
1630			17

1640			17
1650			17
1700			17
800	2	1	1
810	2	1	1
820	2	1	12,15,16
830	1	1	12,13
840	1	1	12,13
850	2	3,5	4,12,13
900	2	14	5,10
910	2	14	5,10,15,16
920	2	14	4,10
930	2	14	4,10
940	2	1	4,12,15,16
950	3	1	4,12
1000	3	7	10
1010	2	1	10
1020	3	7	4,13
1040	2	1	1
1050	9		17
1100	9		17
1110	9		17
1120	9		17
1130	9		17
1140	9		17
1150	9		17
1200	3	1	1
1210	2	1	1
1220	2	1	1
1230	2	1	10
1240	2	1	5,10
1250	2	1	5,10
1300	2	2	4,10
1310	2	1	10
1320	2	1	13
1340	2	2	4,10
1350	2	10	4,10
1400	2	10	4,10
1410	2	10	1,4
1420	2	10	1,4
1430	2	10	1,4
1440	2	10	1,4
1450	2	10	1,4
1500	2	10	1,4
1510	2	10	1,4
1520	2	10	1,4
1530	2	10	1,4
1540	2	1	1

1550	2	1	1
1600	2	1	1
1620	2	1	13
1630	2	12	4,10
1640	2	10	4
1650	2	10	1,4
1700	2	10	1,4
800	2	1	5
810	2	1	5
820	2	1	5,6
830	2	3	5,6
840	2	3	5,6
850	2	1	6
900	1	3	4,7
910	1	3	4,7
920	2	3,4	4,7
930	2	3,5	1
940	2	1	1,12,13
950	2	1	1
1000	2	3,14	4
1010			17
1020			17
1040			17
1050			17
1100			17
1110			17
1120			17
1130			17
1140			17
1150			17
1200			17
1210			17
1220			17
1230			17
1240			17
1250			17
1300			17
1310			17
1320			17
1340			17
1350			17
1400			17
1410			17
1420			17
1430			17
1440			17
1450			17
1500			17
1510			17
1520			17

1530			17
1540			17
1550			17
1600			17
1620			17
1630			17
1640			17
1650			17
1700			17
800	2	1	5
810	2	1	5
820	2	1	5,10,15,16
830	1	1	12,13
840	1	1	12,13
850	2	8	4,10
900	2	1	12,13
910	2	1	10
920	3	9	4,13
930	2	1	10
940	1	1	17
950	2	1	10
1000	2	1	10
1010	4	8,12	4,12,13,15,16
1020	4	8,12	4,12,13,15,16
1040	4	8	4,12,13
1050	4	8	4,12,13
1100	2	9	4,10
1110	2	9	4,10
1120	2	9	4,10
1130	2	9	4,10
1140	2	9	4,10
1150	2	1	10
1200	2	1	10
1210	2	1	1
1220	2	1	1
1230	2	1	10
1240	2	1	10
1250	2	1	5,10
1300	2	1	4,5,10
1310	2	3	4,5,10
1320	2	14	4,10
1340	2	14	4,10
1350	2	10	4,10
1400	2	10	4,10
1410	1	1	10,12,13
1420	2	5,10	4,10
1430			17

1440			17
1450			17
1500			17
1510			17
1520			17
1530			17
1540			17
1550			17
1600			17
1620			17
1630			17
1640			17
1650			17
1700			17
800	5	1	1,7
810	2	3	4
820	5	1	5,10,15,1<br>6
830	5	1	10
840	3	1	12,13
850	3	1	13
900	5	1	4,10
910	5	1	4,10
920	5	1	4,10
930	5	1	4,10
940	5	1	4,10
950	5	1	4,10
1000	5	1	4
1010	10		17
1020	10		17
1040	10		17
1050	9		17
1100	9		17
1110	9		17
1120	9		17
1130	9		17
1140	9		17
1150	9		17
1200	2	1	10
1210	5	1	10
1220	5	14	4,10
1230	5	14	5,10
1240	5	14	4,5,10
1250	5	14	5,10
1300	2	1	1
1310	3	1	13
1320			17
1340			17
1350			17
1400			17

1410			17
1420			17
1430	3	10	4,13
1440	2	2,10	4,10
1450	2	10	4,10
1500	2	10	4,10
1510	3	10	13
1520			17
1530			17
1540			17
1550			17
1600			17
1620			17
1630			17
1640			17
1650			17
1700			17
800	2	1	1
810	2	1	1
820	2	1	1
830	2	1	1
840	2	5	4,7,15,16
850	2	5	4,10,15,16
900	5	5	4,5,10,15,16
910	5	14	4,10
920	5	5,14	4,5,10
930	5	14	4,10
940	2	12	3,4,10
950	2	12	4,10
1000	2	1	4,10,15,16
1010	2	1	4
1020	2	1	1
1030	2	1	1
1040	2	1	1
1050	2	1	1
1100	2	1	1
1110	2	1	1
1120	2	1	4
1130	2	1	1
1140	5	1	10
1200	5	14	4,10
1210	5	14	5,10,15,16
1220	5	1	5,10
1230	5	1	5,10
1240	5	1	5,10
1250	5	14	4,10

1300	1	5	10,13
1310	6	2,10	4,10
1320	6	2,10	4,10
1330	5	10	4,10
1340	5	10	4,10
1350	5	10	4,10
1400	2	3	4,12
1410	2	1	4,10
1420	2	1	4,10
1430	2	1	4,10
1450	2	1	4,10
1500	2	1	4,10
1510	2	1	1
1520	2	1	1
1530	2	1	1
1550	2	1	1
1600	2	1	1
1610	2	1	1
1620	2	1	1
800	2	1	1
810	2	1	1
820	2	1	1
830	1	5	1
840	2	1	1
850	2	5	1
900	2	5,6	4,9
910	2	5	7
920	2	5	7,15,16
930	5	5	5,7,15,16
940	5	5	4,7,15,16
950	3	5	7
1000	3	1	7
1010	3	1	7
1020	3	1	7
1030	3	1	7
1040	2	3,5,6	7,12
1050	9		17
1100	9		17
1110	9		17
1120	3	1	1
1130	3	3,5,6	1
1140	2	1	1
1200	2	1	1
1210	5	1	7
1220	5	1	5,7
1230	5	1	5,7
1240	5	5	5,7
1250	5	5	4,5,7
1300	5	1	7
1310	5	1	7

1320	2	1	1
1330	2	1	1
1340	2	1	1
1350	2	1	15,16
1400	2	1	1
1410	2	1	1
1420	2	1	1
1430	2	1	1
1450	5	9	5,7
1500	5	9	5,7
1510	5	9	5,7
1520	5	10	4,7
1530	5	3,10	4,7
1550	5	10	4,7
1600	5	10	4,7
1610	5	10	4,7
1620	5	10	4,7
800	2	5	12,15,16
810	2	5	12,15,16
820	3	5	4,7,15,16
830	3	1	4,7
840	2	1	7,15,16
850			17
900	3	1	7
910	2	1	4,7
920	2	1	4,7
930	2	1	4,7
940	4	7	4,7,15,16
950	4	7	4,12,15,16
1000	4	7	4,12,15,16
1010	4	7	4,15,16
1020	4	7	4,15,16
1030	4	7	4,12,13,15,16
1040	3	7	4,13
1050	2	1	7
1100	2	1	4,7
1110	2	2	4,7
1120	2	1	4,7
1130	2	1	4,7
1140	5	1	4,7
1200	2	1	4,7
1210	5	1	5,7
1220	5	1	5,7
1230	5	1	5,7
1240	5	1	5,7
1250	2	1	4,7
1300	2	1	4,7

1310	2	1	4,7
1320	2	1	4,7
1330	2	1	4
1340	2	1	4
1350	1	5	4,7,12
1400	2	1	1
1410	2	1	1
1420	2	1	1
1430	2	1	1
1450	2	1	1
1500	2	1	1
1510	2	1	1
1520	2	12	4
1530	2	7	4,10
1550	2	1	1
1600	2	1	1
1610	2	1	1
1620	2	1	1
800	5	1	4,10,15,16
810	5	1	5,10,15,16
820	5	1	5,10,15,16
830	5	1	10
840	5	1	7,15,16
850	3	1	7
900	2	12	4
910	3	1	13
920	2	1	1
930	2	1	1
940	2	1	1
950	2	1	1
1000	2	1	1
1010	2	1	1
1020	2	9	4,7
1030	2	9	4,7
1040	2	9	4,7
1050	3	1	4,7
1100	4	8,12	4,7
1110	4	8	4,7
1120	4	8,12	4,7
1130	2	1	1
1140	2	1	1
1200	2	1	1
1210	5	1	5,7
1220	5	1	5,7
1230	5	1	5,7
1240	3	1	7
1250	3	1	7

1300	4	7,12	4,7,15,16
1310	4	7,12	4,7,12
1320	4	7,12	4,10,12
1330	4	7	4,10,15,16
1340	3	7	4,7
1350	2	1	1
1400	2	1	1
1410	2	1	1
1420	2	1	1
1430	2	1	1
1450	2	1	1
1500	2	1	1
1510	2	1	1
1520	2	1	1
1530	2	1	1
1550	2	1	1
1600	2	1	1
1610	2	1	1
1620	2	1	1
800	1	5	10,15,16
810	1	5	10,15,16
820	2	5,6	4,15,16
830	2	5,6	4,10,15,16
840	5	14	4,5,10,15,16
850	5	14	4,10
900	5	14	4,10
910	2	1	1
920	2	12	4
930	2	12	4
940	2	1	1
950	2	1	1
1000	2	1	1
1010	2	1	1
1020	2	1	4,15,16
1030	2	5	4,15,16
1040	2	5,6	4,10,15,16
1050	3	7	4
1100	3	1	1
1110	3	1	1
1120	3	1	1
1130	3	1	1
1140	3	1	1
1200	3	5	13
1210	5	1	5,10
1220	5	1	5,10
1230	5	1	5,10

1240	5	1	5,10
1250	3	1	1
1300	3	1	1
1310	3	1	1
1320	3	1	1
1330	3	5	13
1340			17
1350			17
1400			17
1410			17
1420			17
1430			17
1450			17
1500			17
1510			17
1520			17
1530			17
1550			17
1600			17
1610			17
1620			17
800			17
810			17
820			17
830	2	3,4	4,7
840	2	3,4	4,7
850	2	3,4	4,7
900	2	10	4,7
910	2	10	4,7
920	2	10	4,7
930	2	10	4,7
940	2	10	4,7
950	2	10	4,7
1000	2	10	4,7
1010	2	10	4,7
1020	2	10	4,7
1030	2	10	4,7
1040	2	10	4,7
1050	2	10	4,7
1100	2	10	4,7
1110	2	10	4,7
1120	2	7	4,7
1130	2	7	4,7
1140	2	7	4,7
1150	2	7	4,7
1210	2	1	1
1220	2	5	4
1230	2	1	1
1240	2	1	1
1250	2	1	1

1300	2	1	1
1310	2	1	1
1320	1	1	1
1330	2	1	1
1340	2	1	1
1350	2	1	1
1400	2	1	1
1410	2	5,6	4,7
1420	4	9	4,7
1430	4	9	4,7
1440	4	9	4,7
1450	3	9	7
1510	5	1	4,7,15,16
1520	5	1	4,7,15,16
1530	5	1	4,7
1540	5	1	4,7
1550	5	1	4,7
1600	2	3,5	4,7
1610	2	1	1
1620	2	1	1
1630	2	1	1
1640	2	1	1
1650	2	1	1
1700	2	1	1
800			17
810			17
820			17
830	3	1	10
840	3	1	10
850	3	1	10
900	3	1	10
910	4	9,12	4,10
920	4	9	4,10
930	4	9,12	4,10
940	4	9,12	4,10
950	4	9	4,10
1000	4	9	4,10
1010	4	9	4,10
1020	4	9	4,10
1030	4	9	4,10
1040	4	9	4,10
1050	4	9	4,10
1100	4	7	4,12,13
1110	4	7	4,10,12,13
1120	3	7	13
1130	2	7	4,10,15,16
1140	2	7	4,10,15,16

1150	2	7	4,10
1210	5	1	10
1220	5	1	4,10
1230	5	1	5,10
1240	2	1	10
1250	2	1	1
1300	2	1	1
1310	2	1	1
1320	1	1	12
1330	2	1	1
1340	2	1	1
1350	2	1	10
1400	5	1	5,10
1410	4	8	4,10
1420	4	8	4,10
1430	4	8	4,10
1440	4	8	4,10,15,16
1450	4	8	4,10
1510	2	1	4
1520	2	1	4
1530	2	1	4
1540	2	1	4
1550	2	1	4
1600	2	1	4,10
1610	2	1	4,10
1620	2	1	4,10
1630	2	1	1
1640	2	1	1
1650	2	1	4,10
1700	2	1	4,10
800			17
810			17
820			17
830	2	5,6	4
840	2	5,6	12
850	2	5,6	4,12
900	1	1	7
910	1	5	4,7
920	1	5	4,7
930	1	5	4,7
940	2	5	4
950	3	5	4,5,7
1000	5	1	5,7
1010	10	13	4,7
1020	10	13	4,7
1030	2	1	7,15,16
1040	1	1	7
1050	3	5	4,7
1100	5	1	7

1110	3	1	7
1120	3	1	2,7
1130	3	1	2,7
1140	3	1	2,7
1150	3	1	2,7
1210	1	5	7
1220	5	1	4
1230	5	1	2,5,7
1240	3	1	2,7
1250	3	1	2,7
1300	3	8	2,4,7
1310	4	7,8	4,7,12
1320	4	7,8	4,12
1330	4	7,8	4,12
1340	4	7,8	4,7
1350	3	10	4,7,15,16
1400	5	1	2,5,7,11
1410	3	1	2,7,11
1420	3	1	2,7,11
1430	10		17
1440	3	1	2,7,11
1450	3	1	2,7,11,15
1510	2	1	2,7,11
1520	1	5	2,7,12
1530	3	1	2,7,11
1540	3	1	2,7,11,15
1550	3	1	2,7,11
1600	5	1	2,7,11,15
1610	3	1	2,7,11,15
1620	3	1	2,7,11
1630	3	1	2,7,11,15
1640	5	1	2,7
1650	5	1	2,7
1700	5	1	2,7
800			17
810			17
820			17
830	3	1	7
840	3	1	7
850			17
900	3	1	3,7
910	3	1	3,7
920	3	1	3,7
930	3	1	3,7
940	3	1	7,12
950			17
1000	3	1	4,7
1010	4	8	4,7
1020	4	8	4,7
1030	4	8	4,7

1040	4	8	4,7
1050	4	8	4,7
1100	5	1	7
1110	3	1	7
1120	3	1	3,7
1130	3	1	3,7
1140	3	1	3,7
1150	3	1	3,7
1210	5	1	3,7
1220	5	1	4
1230	5	1	3,5,7
1240	2	1	3,7
1250	2	5,6	4
1300	2	1	1
1310	3	7	4,12
1320	4	7	12,13
1330	4	7	12
1340	2	7	12,13
1350	3	1	7
1400	5	1	3,5,7,11
1410	5	1	3,7
1420	5	1	3,7,11
1430	3	1	3,7,11
1440	3	1	3,7,11
1450	3	1	3,7,11,15
1510	4	9	4,7,11
1520	4	9	4,7
1530	3	1	3,7,11
1540	2	1	1
1550	2	1	1
1600	2	1	1
1610	2	1	1
1620	2	1	1
1630	2	1	1
1640	5	1	3,7
1650	5	1	3,7
1700	5	1	3,7
800	2	1	1
810	2	1	1
820	2	5	5,6
830	2	3	6
840	2	1	4,5,6
850	2	1	1,6
900	2	1	1,6
910	2	1	1,6
920	2	1	1
930	2	1	1
940	2	1	1
950	2	1	1
1000	2	1	1

1010	2	1	1
1030	2	1	1
1040	2	5,6	4,9
1050	2	1	1
1100	2	1	1
1110	2	1	1
1120	2	1	1
1130	2	1	1
1140	2	1	1
1150	2	1	1
1200	2	1	1
1210	2	1	1
1220	2	1	1
1240	2	5	5,6
1250	2	5	5,6
1300	2	5	5,6
1310	2	1	1
1320	2	1	1
1330	2	1	1
1340	2	1	1
1350	2	1	1
1400	2	1	1
1410	2	1	1
1420	2	1	1
1430	2	1	1
1440	2	1	1
1450	2	1	1
1500	2	3	5
1510	2	1	1
1520	2	1	1
1540	2	8	12
1550	2	1	1
1600	2	1	1
1610	2	1	1
1620	2	1	1
1630	2	1	1
1640	2	1	1
1650	2	1	1
1700	2	1	1
800	2	1	1
810	2	1	1
820	2	1	1
830	2	1	1
840	2	1	1
850	2	5	4,15,16
900	2	5,6	1
910	2	3,4,5	1
920	2	3,4	1
930	2	5	1
940	2	5	4,5,6

950	2	1	1
1000	2	1	1
1010	2	1	1
1030	2	1	1
1040	2	2,3,4	1,4
1050	2	1	1
1100	2	1	1
1110	2	1	1
1120	2	1	1
1130	2	1	1
1140	2	1	1
1150	2	1	1
1200	2	5,6	1,4
1210	2	5,6	7
1220	2	1	7
1240	2	5	5,7
1250	2	5	5,7
1300	2	10	4,7
1310	2	3,10	4,7
1320	2	10	4,7
1330	2	10	4,7
1340	2	10	4,7
1350	2	10	4,7
1400	2	10	4,7
1410	2	10	4,7
1420	2	5,6,10	4
1430	2	10	1,4
1440	2	10	1
1450	2	10	1
1500	2	10	1
1510	2	10	4
1520	2	10	4
1540	2	10	1
1550	2	10	1
1600	2	10	4
1610	2	10	1
1620	2	10	1
1630	2	10	1
1640	2	10	1
1650	2	10	1
1700	2	10	1,4
800	5	1	4,7
810	5	1	5,7
820	5	1	5,7,15
830	5	1	5,7
840	5	5	4,5,7
850	5	14	4,7
900	5	1	7
910	2	1	4,7,15,16
920	2	1	7,15

930	2	1	7,15,16
940	2	1	4,7,15,16
950	2	1	7,15
1000	2	1	4,7
1010	2	1	4,7
1030	1	5	4,7
1040	1	5	7
1050	2	1	1
1100	2	1	1
1110	2	1	1
1120	2	1	1
1130	2	1	1
1140	2	1	1
1150	5	5	4,7
1200	5	14	7
1210	5	14	7
1220	5	14	7
1240	5	14	5,7,15,16
1250	5	14	5,7,15,16
1300	4	7	4,12
1310	4	7	4,7,15,16
1320	3	7	4,12,13
1330	4	7	15,16
1340	3	7	7
1350	5	12	4,7
1400	5	12	4,7
1410	2	5	4,10,11
1420	2	1	4
1430	2	1	1
1440	2	1	4
1450	1	5	12
1500	5	14	7
1510	5	14	7
1520	5	14	7
1540	2	1	7,15,16
1550	2	1	7
1600	2	1	4,15,16
1610	3	1	7,15,16
1620	3	1	7,15,16
1630	3	1	7,15,16
1640	5	1	7
1650	3	1	7
1700	2	1	4,7
800	2	1	1
810	2	1	1
820	2	1	1
830	2	5	1
840	1	5	7,15,16
850	5	1	5,7,15
900	5	1	7

910	5	1	7
920	5	1	7
930	2	1	4,7,15
940	2	1	4,7
950	2	7	4,7
1000	4	7	4,7
1010	4	7	4,12
1030	2	1	4,7,15
1040	2	1	4,7,15
1050	2	1	4,7
1100	2	1	7,11
1110	2	1	4,7,15
1120	2	1	4,7
1130	2	1	7
1140	2	1	7
1150	2	1	7
1200	2	1	7
1210	5	14	7
1220	5	14	7
1240	5	1	5,7,15,16
1250	5	1	5,7,15,16
1300	2	5	4
1310	1	5	7
1320	1	1	7
1330	1	1	7
1340	3	12	4,7
1350	3	2	4,7
1400			17
1410			17
1420			17
1430			17
1440			17
1450			17
1500			17
1510			17
1520			17
1540			17
1550			17
1600			17
1610			17
1620			17
1630			17
1640			17
1650			17
1700			17
800	2	5,10	4,10
810	2	10	4,10
820	2	5,10	4,5,10
830	2	10	4,5,10,15,16

840	2	10	4,5,10
850	2	3,10	4,10,15
900	1	10	4,13
910	2	5,10	1
920	2	1	1
930	2	1	1
940	2	10	1
950	2	1	1
1000	2	10	1
1010	2	3,5,10	10
1030	1	3,10	10,15,16
1040	2	5,10	4,7
1050	5	10	4,7
1100	5	10	4,7
1110	1	10	4,10
1120	2	1	1
1130	2	1	1
1140	2	1	1
1150	2	1	1
1200	2	1	1
1210	2	1	1
1220	2	1	1
1240	2	10	5,7,15,16
1250	2	10	5,7,15,16
1300	2	10	5,7
1310	2	10	4,7
1320	2	10	4,7
1330	2	10	4,7
1340	2	10	4,7
1350	2	10	4,7
1400	2	10	4,7
1410	3	7	4,7,11
1420	3	7	4,13
1430	4	7,10	4,10,15,16
1440	4	7	12
1450	4	7,10	4,10,11
1500	5	10,14	7
1510	5	10,14	7
1520	2	1	1
1540	2	3,10	4
1550	2	10	1
1600	2	5,10	1
1610	2	10	1
1620	2	10	1
1630	2	10	1
1640	2	10	1
1650	2	10	1
1700	2	10	4
800	2	1	1

810	2	1	5,10
820	2	1	5,10,15,16
830	2	1	10
840	2	1	4,10,15
850	2	14	4,10
900	2	7	4,10
910	2	7	4,15,16
920	2	7	4,12
930	2	7	4,10
940	2	14	4,10
950	2	8	4,10
1000	2	8	4,10
1010	2	2,3,4,8	4,10
1030	2	8	4,10
1040	2	8	4,10
1050	3	1	13
1100	3	5	10,13
1110	2	5,14	4,10
1120	2	1	10
1130	2	1	4
1140	2	14	4
1150	2	14	4
1200	2	14	4,13
1210	5	14	4,5,10
1220	5	14	4,5,10
1230	5	14	4,5,10
1250	2	10	4,10
1300	2	10	4,10
1310	2	10	4,10
1320	2	10	4,10
1330	2	10	4,10
1340	2	10	4
1350	2	10	4,10
1400	2	10	4,10
1410	2	2,10	4,10
1420	2	1	1
1430	2	1	1
1440	2	14	4,10
1450	2	1	1
1500	2	1	1
1520	2	1	1
1530	2	1	1
1540	2	1	1
1550	2	1	1
1600	2	3	4,15
1610	2	1	4,15,16
1620	2	1	4
1630	2	1	1
1640	2	1	1

1650	2	1	1
1700	2	3,5	6
800	2	1	1
810	2	1	5,10
820	2	1	13
830	2	1	4
840	2	14	4
850	2	1	1
900	2	1	1
910	2	8	4,10
920	2	8	4,10,15,16
930	2	8	4,10
940	2	8	4
950	2	1	1
1000	2	1	1
1010	2	1	1
1030	2	7	4
1040	2	7	4
1050	2	7	4
1100	2	7	4,12
1110	2	1	1
1120	2	1	1
1130	2	1	1
1140	2	1	1
1150	2	1	1
1200	2	1	1
1210	2	14	4,5,10
1220	2	1	5,10
1230	2	1	5,10
1250	2	1	1
1300	2	1	1
1310	2	1	4
1320	2	2	4,15,16
1330	2	1	1
1340	2	1	1
1350	2	10	4
1400	2	10	4
1410	2	1	1
1420	2	1	1
1430	2	1	1
1440	2	1	1
1450	2	1	1
1500	2	1	1
1520	2	1	1
1530	2	1	1
1540	3	1	4,15,16
1550	3	1	1
1600	7		17
1610	7		17

1620	7		17
1630	7		17
1640	7		17
1650	7		17
1700	7		17
800	2	1	10
810	2	1	5,10
820	2	3,4	4,10,15,16
830	2	1	10
840	2	1	10
850	2	1	10
900	2	1	10
910	2	1	10
920	1	1	12
930	1	1	10
940	1	1	13
950	2	1	10,15,16
1000	2	1	10
1010	2	2,3,4	4,10
1030	2	14	4,13
1040	2	14	4,13
1050	2	1	10
1100	2	1	10
1110	2	1	10
1120	3	1	13
1130	5	1	4,10
1140	5	1	4,10
1150	5	1	10
1200	5	1	10
1210	5	1	4,5,10
1220	5	1	5,10
1230	5	1	5,10
1250	2	1	10
1300	2	1	10
1310	2	1	10
1320	2	1	10
1330	2	1	10
1340	2	1	10
1350	2	1	10
1400	2	1	10
1410	1	1	13
1420	2	1	1
1430	2	1	1
1440	2	1	1
1450	2	1	1
1500	2	1	1
1520	2	1	1
1530	2	1	1
1540	2	14	4

1550	2	14	4
1600	2	5	4,15
1610	2	1	1
1620	2	1	1
1630	2	1	1
1640	2	1	1
1650	2	1	1
1700	2	1	1
