# Supplementary material for: A comparative study of patients’ activities and interactions in a stroke unit before and after reconstruction—The significance of the built environment
Source: PLoS One. 2017 Jul 20;12(7):e0177477. doi: 10.1371/journal.pone.0177477 (PMC5519004; doi:10.1371/journal.pone.0177477)
Supplement: S2 Fig — (PDF) [file pone.0177477.s002.pdf]

| TIME | Location | People | Activity |
|---|---|---|---|
| 800 | 2 | 1 | 1 |
| 810 | 2 | 1 | 1 |
| 820 | 2 | 5 | 1 |
| 830 | 2 | 5 | 4 |
| 840 | 1 | 5 | 4,7,15 |
| 850 | 3 | 5 | 4,7 |
| 900 | 5 | 5 | 5,7,15 |
| 910 | 5 | 1 | 7 |
| 920 | 5 | 14 | 7 |
| 930 | 2 | 5,6 | 4,11 |
| 940 | 2 | 1 | 1 |
| 950 | 2 | 1 | 1 |
| 1000 | 2 | 1 | 1 |
| 1010 | 2 | 1 | 1 |
| 1020 | 2 | 1 | 1 |
| 1030 | 2 | 1 | 1 |
| 1040 | 2 | 1 | 1 |
| 1050 | 2 | 1 | 1 |
| 1100 | 2 | 1 | 1 |
| 1110 | 2 | 1 | 1 |
| 1120 | 2 | 1 | 1 |
| 1130 | 2 | 1 | 1 |
| 1140 | 2 | 1 | 1 |
| 1200 | 2 | 7 | 4 |
| 1210 | 2 | 5,7 | 4,7,9 |
| 1220 | 5 | 5,14 | 5,7,15 |
| 1230 | 5 | 5,14 | 4,7 |
| 1240 | 5 | 5,14 | 4,7 |
| 1250 | 2 | 1 | 4,15 |
| 1300 | 2 | 1 | 1 |
| 1310 | 2 | 1 | 1 |
| 1320 | 2 | 1 | 1 |
| 1330 | 2 | 7,13 | 4,7,9 |
| 1340 | 4 | 7,12,13 | 4,7 |
| 1350 | 4 | 5,7,8 | 4,7,17 |
| 1400 | 3 | 5,7 | 4,7 |
| 1410 | 2 | 1 | 1 |
| 1420 | 2 | 1 | 1 |
| 1430 | 2 | 1 | 1 |
| 1440 | 2 | 1 | 1 |
| 1450 | 2 | 1 | 1 |
| 1500 | 2 | 1 | 1 |
| 1510 | 2 | 1 | 1 |
| 1530 | 2 | 1 | 1 |
| 1540 | 2 | 1 | 1 |
| 1550 | 2 | 1 | 1 |
| 1600 | 2 | 1 | 1 |
| 1610 | 2 | 1 | 1 |
| 1620 | 2 | 1 | 1 |

| | | | |
|---|---|---|---|
| 1630 | 2 | 1 | 1 |
| 1640 | 2 | 10 | 4 |
| 1650 | 2 | 10 | 4 |
| 1700 | 2 | 10 | 4 |
| 800 | 2 | 1 | 1 |
| 810 | 2 | 1 | 1 |
| 820 | 2 | 1 | 1 |
| 830 | 2 | 1 | 1 |
| 840 | 2 | 1 | 1 |
| 850 | 2 | 5 | 1 |
| 900 | 2 | 5 | 1 |
| 910 | 3 | 1 | 7 |
| 920 | 5 | 5 | 4,5,7,15 |
| 930 | 5 | 5 | 5,7 |
| 940 | 4 | 8 | 4,7,15,16 |
| 950 | 4 | 8 | 4,7,15,16 |
| 1000 | 4 | 8 | 4,12 |
| 1010 | 4 | 8 | 4,7 |
| 1020 | 3 | 8 | 7 |
| 1030 | 2 | 1 | 1 |
| 1040 | 2 | 1 | 1 |
| 1050 | 2 | 1 | 1 |
| 1100 | 2 | 1 | 1 |
| 1110 | 2 | 1 | 1 |
| 1120 | 2 | 1 | 1 |
| 1130 | 2 | 5,6 | 7,9 |
| 1140 | 2 | 5,6 | 7,9 |
| 1200 | 5 | 5,14 | 5,7 |
| 1210 | 5 | 14 | 5,7,15 |
| 1220 | 5 | 14 | 7 |
| 1230 | 5 | 5,14 | 4,7 |
| 1240 | 5 | 5,14 | 4,7 |
| 1250 | 5 | 14 | 7 |
| 1300 | 5 | 14 | 7 |
| 1310 | 2 | 1 | 1 |
| 1320 | 2 | 1 | 1 |
| 1330 | 2 | 1 | 1 |
| 1340 | 2 | 1 | 1 |
| 1350 | 2 | 1 | 1 |
| 1400 | 2 | 1 | 1 |
| 1410 | 2 | 3,5 | 7,9 |
| 1420 | 4 | 1 | 1 |
| 1430 | 4 | 9 | 7 |
| 1440 | 3 | 9 | 4,7 |
| 1450 | 3 | 9 | 4,5,7 |
| 1500 | 3 | 5,9 | 4,5,7 |
| 1510 | 3 | 5,9 | 4,7 |
| 1530 | 3 | 1 | 7 |
| 1540 | 3 | 1 | 7 |
| 1550 | 1 | 5,6 | 7,12 |

| | | | |
|---|---|---|---|
| 1600 | 1 | 5,6 | 7 |
| 1610 | 2 | 1 | 1 |
| 1620 | 2 | 1 | 1 |
| 1630 | 2 | 1 | 1 |
| 1640 | 2 | 1 | 1 |
| 1650 | 2 | 1 | 1 |
| 1700 | 2 | 1 | 1 |
| 800 | 2 | 1 | 5,10 |
| 810 | 2 | 5 | 4,5,10 |
| 820 | 2 | 1 | 5,10,15,16 |
| 830 | 2 | 1 | 5,10 |
| 840 | 3 | 14 | 4,12,13 |
| 850 | 3 | 1 | 4,10,15,16 |
| 900 | 3 | 1 | 13 |
| 910 | 3 | 1 | 10 |
| 920 | 3 | 1 | 4,10 |
| 930 | 3 | 1 | 10 |
| 940 | 2 | 1 | 1 |
| 950 | 2 | 1 | 1 |
| 1000 | 2 | 1 | 1 |
| 1010 | 2 | 1 | 1 |
| 1020 | 2 | 1 | 1 |
| 1030 | 2 | 1 | 4,10,15,16 |
| 1040 | 2 | 1 | 4,10,15,16 |
| 1050 | 3 | 14 | 4,10 |
| 1100 | 3 | 14 | 4,10,15,16 |
| 1110 | 3 | 14 | 4,10,15,16 |
| 1120 | 3 | 14 | 4,10 |
| 1130 | 3 | 14 | 4,10 |
| 1140 | 3 | 14 | 4,10 |
| 1200 | 5 | 14 | 4,5,10,15,16 |
| 1210 | 5 | 14 | 4,5,10,15,16 |
| 1220 | 5 | 14 | 4,10 |
| 1230 | 5 | 5,14 | 4,10 |
| 1240 | 5 | 5,14 | 4,10 |
| 1250 | 5 | 14 | 4,10 |
| 1300 | 5 | 14 | 4,10 |
| 1310 | 3 | 1 | 13 |
| 1320 | 3 | 3,14 | 4,12 |
| 1330 | 3 | 12 | 4,12,13 |
| 1340 | 3 | 14 | 4,10 |

| | | | |
|---|---|---|---|
| 1350 | 3 | 14 | 4,10,15,16 |
| 1400 | 3 | 14 | 4,10,15,16 |
| 1410 | 3 | 14 | 4,10,15,16 |
| 1420 | 3 | 14 | 4,10,15,16 |
| 1430 | 3 | 14 | 4,10 |
| 1440 | 3 | 14 | 4,10,15,16 |
| 1450 | 3 | 14 | 4,5,10 |
| 1500 | 3 | 14 | 4,10,15,16 |
| 1510 | 3 | 14 | 4,10 |
| 1530 | 3 | 14 | 4,10 |
| 1540 | 2 | 1 | 4,10 |
| 1550 | 3 | 14 | 4,10 |
| 1600 | 3 | 14 | 4,10 |
| 1610 | 3 | 14 | 4,10 |
| 1620 | 3 | 14 | 4,10 |
| 1630 | 3 | 14 | 4,13 |
| 1640 | 2 | 1 | 4 |
| 1650 | 2 | 1 | 4,15,16 |
| 1700 | 2 | 1 | 1 |
| 800 | 2 | 1 | 5,10 |
| 810 | 2 | 1 | 4,10 |
| 820 | 2 | 1 | 5,10,15 |
| 830 | 2 | 1 | 4,10 |
| 840 | 2 | 1 | 4,10 |
| 850 | 2 | 1 | 4,10,15 |
| 900 | 2 | 1 | 4,10 |
| 910 | 2 | 1 | 4,10 |
| 920 | 2 | 1 | 1 |
| 930 | 2 | 1 | 4,10 |
| 940 | 2 | 1 | 4,10 |
| 950 | 2 | 1 | 4,10 |
| 1000 | 2 | 1 | 4,10,15,16 |
| 1010 | 2 | 1 | 4,10 |
| 1020 | 2 | 1 | 4,12 |
| 1030 | 2 | 3 | 4,10 |
| 1040 | 2 | 1 | 4,5,15,16 |
| 1050 | 2 | 8 | 4 |
| 1100 | 3 | 7 | 4,13,15,16 |
| 1110 | 2 | 1 | 4,10,15,16 |
| 1120 | 3 | 7 | 4,13 |
| 1130 | 2 | 1 | 4,10 |

| | | | |
|---|---|---|---|
| 1140 | 2 | 1 | 4,10 |
| 1200 | 2 | 1 | 4,5,10 |
| 1210 | 2 | 1 | 10 |
| 1220 | 2 | 1 | 4,13 |
| 1230 | 2 | 1 | 4,15,16 |
| 1240 | 2 | 1 | 4,15,16 |
| 1250 | 2 | 1 | 4,15 |
| 1300 | 2 | 5 | 4 |
| 1310 | 2 | 1 | 4,10 |
| 1320 | 2 | 1 | 4,10 |
| 1330 | 2 | 10 | 4,10,15,16 |
| 1340 | 10 | | 17 |
| 1350 | 10 | 2,10 | 4,10 |
| 1400 | 2 | 5,6,10 | 4,13 |
| 1410 | | | 17 |
| 1420 | | | 17 |
| 1430 | | | 17 |
| 1440 | | | 17 |
| 1450 | | | 17 |
| 1500 | | | 17 |
| 1510 | | | 17 |
| 1530 | | | 17 |
| 1540 | | | 17 |
| 1550 | | | 17 |
| 1600 | | | 17 |
| 1610 | | | 17 |
| 1620 | | | 17 |
| 1630 | | | 17 |
| 1640 | | | 17 |
| 1650 | | | 17 |
| 1700 | | | 17 |
| 800 | 2 | 1 | 5,7 |
| 810 | 2 | 1 | 4,7 |
| 820 | 2 | 1 | 5,7,15 |
| 830 | 2 | 1 | 4,7 |
| 840 | 2 | 1 | 4,7 |
| 850 | 2 | 1 | 7,15,16 |
| 900 | 1 | 1 | 7,11 |
| 910 | 2 | 1 | 4,7 |
| 920 | 1 | 1 | 7,11 |
| 930 | 2 | 1 | 4,7 |
| 940 | 2 | 1 | 4,7 |
| 950 | 2 | 1 | 4,7 |
| 1000 | 2 | 1 | 4,7 |
| 1010 | 2 | 1 | 4,7 |
| 1020 | 2 | 1 | 4,6 |
| 1030 | 8 | | 17 |
| 1040 | 8 | 1 | 17 |
| 1050 | 8 | | 17 |

| | | | |
|---|---|---|---|
| 1100 | 8 | | 17 |
| 1110 | 8 | | 17 |
| 1120 | 8 | | 17 |
| 1130 | 8 | | 17 |
| 1140 | 2 | 1 | 4 |
| 1200 | 2 | 1 | 4,5,10 |
| 1210 | 1 | 1 | 7,12 |
| 1220 | 2 | 1 | 4,10 |
| 1230 | 2 | 10 | 4,10 |
| 1240 | 2 | 10 | 4,10 |
| 1250 | 2 | 10 | 4,10 |
| 1300 | 2 | 10 | 4,10 |
| 1310 | 2 | 10 | 4,10 |
| 1320 | 2 | 10 | 4,10 |
| 1330 | 2 | 10 | 4,10 |
| 1340 | 2 | 10 | 4,10 |
| 1350 | 2 | 10 | 4,10 |
| 1400 | 2 | 10 | 4,10 |
| 1410 | 2 | 10,13 | 4,10,15,16 |
| 1420 | 2 | 10 | 4,10 |
| 1430 | 2 | 10 | 4,7 |
| 1440 | 2 | 10 | 4,7 |
| 1450 | 2 | 10 | 4,7 |
| 1500 | 2 | 10 | 4,7 |
| 1510 | 2 | 10 | 4,7 |
| 1530 | 2 | 1 | 7 |
| 1540 | 2 | 1 | 4,7 |
| 1550 | 2 | 1 | 4 |
| 1600 | 2 | 1 | 4 |
| 1610 | 2 | 1 | 4 |
| 1620 | 2 | 1 | 4 |
| 1630 | 2 | 1 | 1 |
| 1640 | 2 | 1 | 1 |
| 1650 | 2 | 1 | 1 |
| 1700 | 2 | 5 | 4,7,9 |
| 800 | 2 | 1 | 1 |
| 810 | 2 | 1 | 1 |
| 820 | 2 | 1 | 1 |
| 830 | 2 | 3 | 4,5,6 |
| 840 | 2 | 1 | 6,15,16 |
| 850 | | | 17 |
| 900 | 2 | 1 | 1 |
| 910 | 2 | 1 | 1 |
| 920 | 1 | 1 | 10 |
| 930 | 2 | 1 | 10,15,16 |
| 940 | 2 | 1 | 6 |
| 950 | 2 | 1 | 6 |
| 1000 | 2 | 1 | 4,6,15,16 |
| 1010 | 2 | 1 | 6 |

| | | | |
|---|---|---|---|
| 1020 | 2 | 1 | 6 |
| 1030 | 2 | 1 | 6 |
| 1040 | 2 | 1 | 1 |
| 1050 | 2 | 1 | 1 |
| 1100 | 2 | 1 | 1 |
| 1110 | 2 | 5,6 | 1,4 |
| 1120 | 2 | 1 | 15,16 |
| 1130 | 2 | 1 | 1 |
| 1140 | 2 | 1 | 1 |
| 1200 | 2 | 1 | 5,6 |
| 1210 | 2 | 1 | 5,15,16 |
| 1220 | 2 | 1 | 6 |
| 1230 | 2 | 1 | 4,6 |
| 1240 | 2 | 1 | 6 |
| 1250 | 2 | 1 | 6,15 |
| 1300 | 2 | 1 | 6 |
| 1310 | 2 | 1 | 6,15,16 |
| 1320 | 2 | 1 | 6,15,16 |
| 1330 | 2 | 1 | 6 |
| 1340 | 3 | 1 | 13,15,16 |
| 1350 | 3 | 1 | 13,15,16 |
| 1400 | 3 | 1 | 13 |
| 1410 | 2 | 1 | 10,15,16 |
| 1420 | 2 | 1 | 4,6,15,16 |
| 1430 | 2 | 1 | 4,6 |
| 1440 | 2 | 1 | 4,7 |
| 1450 | 2 | 14 | 4,6 |
| 1500 | 2 | 1 | 4,6 |
| 1510 | 2 | 1 | 4,6 |
| 1530 | 2 | 1 | 4,6 |
| 1540 | 2 | 1 | 4,6 |
| 1550 | 2 | 1 | 4,6 |
| 1600 | 2 | 1 | 4 |
| 1610 | 2 | 1 | 4,6 |
| 1620 | 2 | 1 | 4,6 |
| 1630 | 2 | 1 | 1 |
| 1640 | 2 | 1 | 1 |
| 1650 | 2 | 1 | 4,10 |
| 1700 | 2 | 5 | 4,5,10 |
| 800 | 1 | 5 | 7,15,16 |
| 810 | 3 | 1 | 7 |
| 820 | 3 | 5 | 5,7 |
| 830 | 3 | 5 | 5,7,15 |
| 840 | 3 | 1 | 7 |
| 850 | 3 | 1 | 7 |
| 900 | 3 | 1 | 5,7,15 |
| 910 | 3 | 1 | 7 |
| 920 | 3 | 1 | 7 |
| 930 | 3 | 1 | 7 |
| 940 | 3 | 1 | 7 |

| | | | |
|---|---|---|---|
| 950 | 2 | 1 | 7 |
| 1000 | 2 | 1 | 1 |
| 1010 | 2 | 1 | 1 |
| 1030 | 2 | 1 | 1 |
| 1040 | 2 | 1 | 1 |
| 1050 | 2 | 3 | 4 |
| 1100 | 2 | 1 | 1 |
| 1110 | 2 | 1 | 1 |
| 1120 | 2 | 1 | 1 |
| 1130 | 2 | 1 | 1 |
| 1140 | 2 | 1 | 1 |
| 1150 | 2 | 1 | 1 |
| 1200 | 2 | 1 | 1 |
| 1210 | 2 | 1 | 1 |
| 1220 | 2 | 5 | 4,5,6 |
| 1230 | 2 | 3,5 | 1 |
| 1240 | 2 | 1 | 1 |
| 1250 | 2 | 1 | 1 |
| 1300 | 2 | 1 | 1 |
| 1320 | 2 | 1 | 1 |
| 1330 | 2 | 1 | 1 |
| 1340 | 2 | 1 | 1 |
| 1350 | 2 | 1 | 1 |
| 1400 | 2 | 5 | 1 |
| 1410 | 2 | 1 | 7 |
| 1420 | 2 | 1 | 7 |
| 1430 | 2 | 9 | 4,5,7 |
| 1440 | 2 | 3,5,9 | 6 |
| 1450 | 9 | | 17 |
| 1500 | 9 | | 17 |
| 1510 | 9 | | 17 |
| 1520 | 9 | | 17 |
| 1530 | 9 | | 17 |
| 1540 | 9 | | 17 |
| 1550 | 9 | | 17 |
| 1600 | 9 | | 17 |
| 1620 | 2 | 5,6 | 1 |
| 1630 | 2 | 1 | 1 |
| 1640 | 2 | 1 | 1 |
| 1650 | 2 | 1 | 1 |
| 1700 | 2 | 1 | 1 |
| 800 | 2 | 1 | 1 |
| 810 | 2 | 1 | 1 |
| 820 | 2 | 1 | 1 |
| 830 | 2 | 1 | 1 |
| 840 | 2 | 1 | 1 |
| 850 | 2 | 5,6 | 1 |
| 900 | 2 | 5 | 1 |
| 910 | 2 | 1 | 1 |
| 920 | 2 | 1 | 1 |

| | | | |
|---|---|---|---|
| 930 | 2 | 1 | 1 |
| 940 | 2 | 1 | 1 |
| 950 | 2 | 8,9 | 4 |
| 1000 | 2 | 5 | 4,5,10 |
| 1010 | 2 | 1 | 12 |
| 1030 | 10 | | 17 |
| 1040 | 10 | | 17 |
| 1050 | 5 | 1 | 10 |
| 1100 | 5 | 1 | 10 |
| 1110 | 5 | 1 | 10 |
| 1120 | 5 | 1 | 10 |
| 1130 | 5 | 1 | 10 |
| 1140 | 5 | 1 | 10 |
| 1150 | 2 | 1 | 1 |
| 1200 | 2 | 1 | 1 |
| 1210 | 2 | 1 | 5,10 |
| 1220 | 2 | 1 | 5,10 |
| 1230 | 2 | 1 | 5,10 |
| 1240 | 2 | 1 | 1 |
| 1250 | 2 | 1 | 1 |
| 1300 | 2 | 1 | 1 |
| 1320 | 2 | 1 | 1 |
| 1330 | 2 | 1 | 1 |
| 1340 | 2 | 1 | 1 |
| 1350 | 2 | 1 | 1 |
| 1400 | 2 | 1 | 1 |
| 1410 | 2 | 1 | 1 |
| 1420 | 2 | 1 | 1 |
| 1430 | 2 | 1 | 1 |
| 1440 | 2 | 1 | 10 |
| 1450 | 3 | 1 | 13 |
| 1500 | 2 | 1 | 1 |
| 1510 | 2 | 1 | 1 |
| 1520 | 2 | 10 | 12,13 |
| 1530 | 2 | 10 | 13 |
| 1540 | 9 | | 17 |
| 1550 | 9 | | 17 |
| 1600 | 9 | | 17 |
| 1620 | 9 | | 17 |
| 1630 | 9 | | 17 |
| 1640 | 9 | | 17 |
| 1650 | 9 | | 17 |
| 1700 | 9 | | 17 |
| 800 | 2 | 5 | 6 |
| 810 | 2 | 1 | 5,6,15 |
| 820 | 2 | 1 | 6 |
| 830 | 2 | 1 | 5,6,15 |
| 840 | 1 | 5,6 | 7,15 |
| 850 | 1 | 3 | 7,15 |
| 900 | 2 | 1 | 1 |

| | | | |
|---|---|---|---|
| 910 | 2 | 1 | 1 |
| 920 | 2 | 1 | 1 |
| 930 | 2 | 1 | 1 |
| 940 | 2 | 1 | 1 |
| 950 | 2 | 2,3 | 4 |
| 1000 | 2 | 7 | 4 |
| 1010 | 2 | 7 | 4,15,16 |
| 1030 | 8 | | 17 |
| 1040 | 8 | | 17 |
| 1050 | 8 | | 17 |
| 1100 | 8 | | 17 |
| 1110 | 8 | | 17 |
| 1120 | 8 | | 17 |
| 1130 | 2 | 3,5 | 4 |
| 1140 | 2 | 5 | 4,9 |
| 1150 | 2 | 1 | 1 |
| 1200 | 2 | 2 | 4,15,16 |
| 1210 | 2 | 2 | 4,9 |
| 1220 | 2 | 3 | 4,5,6 |
| 1230 | 2 | 1 | 5,6 |
| 1240 | 2 | 1 | 6 |
| 1250 | 2 | 1 | 1 |
| 1300 | 2 | 1 | 1 |
| 1320 | 2 | 1 | 1 |
| 1330 | 2 | 1 | 1 |
| 1340 | 9 | | 17 |
| 1350 | 9 | | 17 |
| 1400 | 9 | | 17 |
| 1410 | 9 | | 17 |
| 1420 | 9 | | 17 |
| 1430 | 2 | 8 | 9,11,15 |
| 1440 | 2 | 8 | 4,15 |
| 1450 | 2 | 1 | 1 |
| 1500 | 2 | 1 | 1 |
| 1510 | 2 | 10 | 4 |
| 1520 | 2 | 10 | 4 |
| 1530 | 2 | 10 | 4 |
| 1540 | 2 | 10 | 4 |
| 1550 | 2 | 10 | 4 |
| 1600 | 2 | 10 | 4 |
| 1620 | 2 | 10 | 4 |
| 1630 | 2 | 10 | 4 |
| 1640 | 2 | 10 | 4 |
| 1650 | 2 | 10 | 4 |
| 1700 | 2 | 10 | 4 |
| 800 | 2 | 1 | 6 |
| 810 | 2 | 1 | 6 |
| 820 | 2 | 1 | 6 |
| 830 | 2 | 1 | 6 |
| 840 | 2 | 3,5 | 4,15 |

| | | | |
|---|---|---|---|
| 850 | 2 | 3,5 | 4,6 |
| 900 | 3 | 1 | 7 |
| 910 | 3 | 3 | 4,7 |
| 920 | 2 | 7 | 4,15 |
| 930 | 2 | 7 | 4,9 |
| 940 | 2 | 7 | 4 |
| 950 | 2 | 1 | 1 |
| 1000 | 2 | 8 | 4 |
| 1010 | 2 | 8 | 4 |
| 1030 | 2 | 1 | 1 |
| 1040 | 2 | 1 | 1 |
| 1050 | 2 | 5 | 1 |
| 1100 | 1 | 1 | 7 |
| 1110 | 2 | 5 | 4,10 |
| 1120 | 2 | 5 | 4 |
| 1130 | 2 | 1 | 1 |
| 1140 | 2 | 3 | 1 |
| 1150 | 2 | 1 | 1 |
| 1200 | 2 | 1 | 1 |
| 1210 | 2 | 1 | 4,6 |
| 1220 | 2 | 1 | 5,6 |
| 1230 | 2 | 1 | 5,6 |
| 1240 | 2 | 1 | 1 |
| 1250 | 2 | 1 | 1 |
| 1300 | 2 | 1 | 1 |
| 1320 | 2 | 1 | 1 |
| 1330 | 2 | 1 | 1 |
| 1340 | 2 | 1 | 1 |
| 1350 | 2 | 1 | 1 |
| 1400 | 2 | 1 | 1 |
| 1410 | 2 | 1 | 1 |
| 1420 | 2 | 1 | 1 |
| 1430 | 2 | 1 | 1 |
| 1440 | 2 | 1 | 1 |
| 1450 | 1 | 3,5 | 7,12 |
| 1500 | 2 | 1 | 1 |
| 1510 | 2 | 1 | 1 |
| 1520 | 2 | 1 | 1 |
| 1530 | 2 | 1 | 1 |
| 1540 | 2 | 1 | 1 |
| 1550 | 2 | 1 | 1 |
| 1600 | 2 | 1 | 1 |
| 1620 | 2 | 1 | 1 |
| 1630 | 2 | 1 | 1 |
| 1640 | 2 | 1 | 1 |
| 1650 | 2 | 1 | 1 |
| 1700 | 2 | 1 | 1 |
| 800 | 3 | 1 | 7 |
| 810 | 3 | 1 | 7 |
| 820 | 3 | 1 | 7 |

| | | | |
|---|---|---|---|
| 830 | 3 | 1 | 7,15 |
| 840 | 3 | 1 | 1 |
| 850 | 2 | 1 | 5,7 |
| 900 | 2 | 1 | 4,7,15 |
| 910 | 2 | 3 | 4,7 |
| 920 | 2 | 1 | 7 |
| 930 | 2 | 1 | 4,7,15 |
| 940 | 2 | 1 | 7 |
| 950 | 2 | 1 | 7 |
| 1000 | 2 | 1 | 7 |
| 1010 | 2 | 1 | 7 |
| 1030 | 2 | 1 | 1 |
| 1040 | 2 | 1 | 1 |
| 1050 | 2 | 1 | 1 |
| 1100 | 2 | 1 | 1 |
| 1110 | 2 | 3,4 | 4,15 |
| 1120 | 2 | 1 | 1 |
| 1130 | 2 | 1 | 1 |
| 1140 | 2 | 3 | 4 |
| 1150 | 2 | 1 | 1 |
| 1200 | 2 | 3,5 | 4,7,9,15 |
| 1210 | 3 | 3 | 4,5,7 |
| 1220 | 3 | 3 | 5,7 |
| 1230 | 3 | 1 | 7 |
| 1240 | 3 | 1 | 7 |
| 1250 | 3 | 1 | 1 |
| 1300 | 3 | 1 | 7 |
| 1320 | 3 | 3 | 7 |
| 1330 | 3 | 3 | 7 |
| 1340 | 3 | 3 | 7 |
| 1350 | 3 | 1 | 7 |
| 1400 | 3 | 1 | 7 |
| 1410 | 3 | 1 | 7,15,16 |
| 1420 | 3 | 1 | 7,15,16 |
| 1430 | 2 | 10 | 4 |
| 1440 | 2 | 10 | 4 |
| 1450 | 2 | 10 | 4 |
| 1500 | 2 | 5,10 | 4 |
| 1510 | 2 | 10 | 4 |
| 1520 | 2 | 1 | 1 |
| 1530 | 2 | 1 | 1 |
| 1540 | 2 | 1 | 1 |
| 1550 | 2 | 1 | 1 |
| 1600 | 2 | 1 | 1 |
| 1620 | 2 | 1 | 15,16 |
| 1630 | 2 | 10 | 4 |
| 1640 | 2 | 10 | 4 |
| 1650 | 2 | 10 | 4 |
| 1700 | 2 | 10 | 4 |
| 800 | | | 17 |

| | | | |
|---|---|---|---|
| 810 | 2 | 1 | 10,15,16 |
| 820 | 2 | 1 | 4,10,15,16 |
| 830 | 2 | 3 | 4,10 |
| 840 | 2 | 1 | 4,10 |
| 850 | 2 | 1 | 4,10,15,16 |
| 900 | 2 | 1 | 4 |
| 910 | 2 | 5 | 4 |
| 920 | 2 | 1 | 4 |
| 930 | 2 | 1 | 4,15,16 |
| 940 | 2 | 1 | 4 |
| 950 | 2 | 1 | 4,6 |
| 1000 | 2 | 1 | 13 |
| 1010 | 2 | 1 | 4,10 |
| 1030 | 2 | 2,3 | 4,10 |
| 1040 | 2 | 1 | 4,10 |
| 1050 | 2 | 1 | 4 |
| 1100 | 2 | 10 | 4,10 |
| 1110 | 2 | 10 | 4,10 |
| 1120 | 2 | 10 | 4,10,15,16 |
| 1130 | 2 | 10 | 10 |
| 1140 | 2 | 10 | 4,10,15,16 |
| 1150 | 10 | | 17 |
| 1200 | 10 | | 17 |
| 1210 | 10 | | 17 |
| 1220 | 10 | | 17 |
| 1230 | 10 | | 17 |
| 1240 | 2 | 1 | 1 |
| 1250 | 2 | 10 | 4,10 |
| 1300 | 2 | 10 | 4 |
| 1320 | 2 | 5,10 | 4 |
| 1330 | 2 | 10 | 4 |
| 1340 | 2 | 10 | 4 |
| 1350 | 2 | 10 | 4 |
| 1400 | 2 | 10 | 4 |
| 1410 | 2 | 3,10 | 4 |
| 1420 | 9 | | 17 |
| 1430 | 2 | 10 | 4 |
| 1440 | 3 | 1 | 13 |
| 1450 | 2 | 1 | 4,10 |
| 1500 | 2 | 1 | 4,10 |
| 1510 | 2 | 1 | 4 |
| 1520 | 2 | 1 | 4,10 |
| 1530 | 2 | 1 | 4 |
| 1540 | 2 | 1 | 4 |
| 1550 | 2 | 1 | 12 |
| 1600 | 2 | 1 | 4,10 |

| | | | |
|---|---|---|---|
| 1620 | 2 | 3 | 4,10 |
| 1630 | 2 | 1 | 4 |
| 1640 | | | 17 |
| 1650 | 2 | 1 | 4 |
| 1700 | 2 | 1 | 4 |
| 800 | 2 | 1 | 5,10 |
| 810 | 2 | 1 | 4,5,10 |
| 820 | 2 | 1 | 10 |
| 830 | 2 | 1 | 4,10 |
| 840 | 2 | 1 | 4,10 |
| 850 | 2 | 1 | 4,10 |
| 900 | 2 | 1 | 4,10 |
| 910 | 2 | 1 | 1 |
| 920 | 2 | 1 | 4,12 |
| 930 | 1 | 1 | 12 |
| 940 | 2 | 1 | 4,12 |
| 950 | 2 | 1 | 4,10 |
| 1000 | 2 | 1 | 4,10 |
| 1010 | 2 | 1 | 4,10 |
| 1020 | 2 | 1 | 4,10 |
| 1030 | 2 | 14 | 4,10 |
| 1040 | 2 | 14 | 4,10 |
| 1050 | 2 | 14 | 4,10 |
| 1100 | 2 | 8 | 4,10 |
| 1110 | 2 | 8 | 4,10 |
| 1120 | 2 | 14 | 4,10 |
| 1130 | 2 | 1 | 10 |
| 1140 | 2 | 1 | 10 |
| 1150 | 2 | 8 | 4,10 |
| 1200 | 2 | 8 | 4,10 |
| 1210 | 2 | 1 | 5,10 |
| 1220 | 2 | 1 | 4,10 |
| 1240 | 2 | 1 | 1 |
| 1250 | 2 | 1 | 1 |
| 1300 | 2 | 1 | 1 |
| 1310 | 2 | 1 | 1 |
| 1320 | 2 | 1 | 1 |
| 1330 | 2 | 1 | 4,10 |
| 1340 | 2 | 1 | 4,10 |
| 1350 | 2 | 1 | 4,10 |
| 1400 | 2 | 14 | 4,10 |
| 1410 | 2 | 12 | 4,10 |
| 1420 | 2 | 1 | 10 |
| 1430 | 2 | 1 | 10 |
| 1440 | 2 | 5,6 | 4,15,16 |
| 1450 | 2 | 1 | 13 |
| 1500 | 2 | 1 | 10 |
| 1510 | 2 | 1 | 10 |
| 1520 | 2 | 1 | 10 |
| 1530 | 2 | 1 | 1 |

| | | | |
|---|---|---|---|
| 1540 | 3 | 1 | 13 |
| 1550 | 2 | 1 | 10 |
| 1600 | 2 | 1 | 10 |
| 1620 | 2 | 1 | 10 |
| 1630 | 2 | 1 | 10 |
| 1640 | 2 | 1 | 10 |
| 1650 | 2 | 1 | 10 |
| 1700 | 2 | 1 | 10 |
| 800 | 2 | 1 | 5,10 |
| 810 | 2 | 1 | 5,10 |
| 820 | 1 | 1 | 12 |
| 830 | 2 | 1 | 4,10,15,16 |
| 840 | 2 | 1 | 10 |
| 850 | 2 | 1 | 1 |
| 900 | 2 | 1 | 1 |
| 910 | 2 | 1 | 1 |
| 920 | 3 | 1 | 13 |
| 930 | 2 | 7 | 4,10 |
| 940 | 2 | 7,12 | 4,15,16 |
| 950 | 2 | 7 | 4 |
| 1000 | 2 | 7 | 4 |
| 1010 | 2 | 8,12 | 4,10 |
| 1020 | 2 | 8,12 | 4,10 |
| 1030 | 2 | 8 | 4,10 |
| 1040 | 2 | 8 | 4,10 |
| 1050 | 2 | 8 | 4,10 |
| 1100 | 5 | 2,10,13 | 4,10 |
| 1110 | 5 | 10 | 4,10 |
| 1120 | 2 | 1 | 10 |
| 1130 | 2 | 14 | 4,10 |
| 1140 | 2 | 1 | 10 |
| 1150 | 2 | 1 | 12 |
| 1200 | 2 | 1 | 5,10 |
| 1210 | 2 | 1 | 5,10 |
| 1220 | 2 | 1 | 10 |
| 1240 | 2 | 1 | 1 |
| 1250 | 2 | 1 | 1 |
| 1300 | 2 | 1 | 1 |
| 1310 | 2 | 5 | 4,10 |
| 1320 | 1 | 1 | 12 |
| 1330 | 2 | 1 | 1 |
| 1340 | 2 | 1 | 1 |
| 1350 | 2 | 1 | 1 |
| 1400 | 2 | 12 | 4,10 |
| 1410 | 2 | 1 | 1 |
| 1420 | | | 17 |
| 1430 | | | 17 |
| 1440 | | | 17 |
| 1450 | | | 17 |

| | | | |
|---|---|---|---|
| 1500 | | | 17 |
| 1510 | | | 17 |
| 1520 | | | 17 |
| 1530 | | | 17 |
| 1540 | | | 17 |
| 1550 | | | 17 |
| 1600 | | | 17 |
| 1620 | | | 17 |
| 1630 | | | 17 |
| 1640 | | | 17 |
| 1650 | | | 17 |
| 1700 | | | 17 |
| 800 | 2 | 1 | 12,13 |
| 810 | 2 | 3 | 5,10 |
| 820 | 2 | 1 | 5,10 |
| 830 | 2 | 1 | 5,10,15,16 |
| 840 | 2 | 1 | 1 |
| 850 | 2 | 1 | 1 |
| 900 | 2 | 1 | 1 |
| 910 | 2 | 1 | 1 |
| 920 | 2 | 1 | 1 |
| 930 | 2 | 1 | 1 |
| 940 | 2 | 1 | 1 |
| 950 | 2 | 1 | 1 |
| 1000 | 2 | 1 | 1 |
| 1010 | 2 | 1 | 1 |
| 1020 | 2 | 1 | 1 |
| 1030 | 1 | 1 | 13 |
| 1040 | 2 | 1 | 10 |
| 1050 | 2 | 1 | 4,10 |
| 1100 | 2 | 1 | 4,15,16 |
| 1110 | 3 | 2 | 13 |
| 1120 | 2 | 1 | 4,10 |
| 1130 | 2 | 14 | 4,12 |
| 1140 | 2 | 1 | 1 |
| 1150 | 2 | 7 | 4,10 |
| 1200 | 2 | 1 | 4,5,10 |
| 1210 | 2 | 1 | 5,10 |
| 1220 | 2 | 1 | 10 |
| 1240 | 2 | 1 | 1 |
| 1250 | 2 | 1 | 1 |
| 1300 | 2 | 1 | 10 |
| 1310 | 2 | 2 | 4,10 |
| 1320 | 2 | 1 | 4,10 |
| 1330 | 2 | 1 | 1 |
| 1340 | 2 | 1 | 1 |
| 1350 | 2 | 1 | 1 |
| 1400 | 2 | 1 | 1 |
| 1410 | 2 | 10 | 4 |

| | | | |
|---|---|---|---|
| 1420 | 2 | 1 | 4,13 |
| 1430 | 2 | 1 | 1 |
| 1440 | 1 | 1 | 13 |
| 1450 | 2 | 1 | 4,15,16 |
| 1500 | 2 | 1 | 1 |
| 1510 | 2 | 1 | 1 |
| 1520 | 2 | 1 | 1 |
| 1530 | 2 | 1 | 13 |
| 1540 | 3 | 1 | 13 |
| 1550 | 2 | 1 | 1 |
| 1600 | 2 | 2 | 1 |
| 1620 | 2 | 1 | 1 |
| 1630 | 2 | 1 | 1 |
| 1640 | 2 | 1 | 1 |
| 1650 | 2 | 10 | 12 |
| 1700 | 2 | 10 | 12,13 |
| 800 | 2 | 1 | 1 |
| 810 | 2 | 1 | 1 |
| 820 | 2 | 1 | 1 |
| 830 | 2 | 5,6 | 4,15,16 |
| 840 | 2 | 5,6 | 4,15,16 |
| 850 | 2 | 3,5,6 | 4 |
| 900 | 2 | 1 | 1 |
| 910 | 2 | 1 | 1 |
| 920 | 2 | 1 | 1 |
| 930 | 2 | 1 | 1 |
| 940 | 2 | 1 | 1 |
| 950 | 2 | 1 | 1 |
| 1000 | 2 | 1 | 1 |
| 1010 | 2 | 1 | 1 |
| 1020 | 2 | 1 | 1 |
| 1030 | 2 | 1 | 1 |
| 1040 | 2 | 2,13 | 1 |
| 1050 | 2 | 1 | 1 |
| 1100 | 2 | 1 | 1 |
| 1110 | 2 | 1 | 1 |
| 1120 | 2 | 1 | 1 |
| 1130 | 2 | 1 | 1 |
| 1140 | 2 | 1 | 1 |
| 1150 | 2 | 1 | 1 |
| 1200 | 2 | 1 | 1 |
| 1210 | 1 | 1 | 1 |
| 1220 | 2 | 1 | 1 |
| 1240 | 2 | 1 | 1 |
| 1250 | 2 | 1 | 1 |
| 1300 | 2 | 1 | 1 |
| 1310 | 2 | 1 | 1 |
| 1320 | 2 | 1 | 1 |
| 1330 | 2 | 1 | 1 |
| 1340 | 2 | 1 | 1 |

| | | | |
|---|---|---|---|
| 1350 | 2 | 1 | 1 |
| 1400 | 2 | 5,6 | 1 |
| 1410 | 2 | 5,6 | 1 |
| 1420 | 2 | 1 | 1 |
| 1430 | 2 | 1 | 1 |
| 1440 | 2 | 1 | 1 |
| 1450 | 2 | 1 | 1 |
| 1500 | 2 | 1 | 1 |
| 1510 | 2 | 1 | 1 |
| 1520 | 2 | 1 | 1 |
| 1530 | 2 | 1 | 1 |
| 1540 | 2 | 1 | 1 |
| 1550 | 2 | 1 | 1 |
| 1600 | 2 | 10 | 1 |
| 1620 | 2 | 10 | 1 |
| 1630 | 2 | 10 | 1 |
| 1640 | 2 | 1 | 1 |
| 1650 | 2 | 10 | 1 |
| 1700 | 2 | 10 | 1 |
| 800 | 2 | 1 | 5,10,15,16 |
| 810 | 2 | 1 | 5,10,15,16 |
| 820 | 2 | 1 | 5,10,15,16 |
| 830 | 1 | 1 | 12,15,16 |
| 840 | 1 | 1 | 12,15,16 |
| 850 | 2 | 1 | 12 |
| 900 | 3 | 1 | 10 |
| 910 | 5 | 1 | 4,10 |
| 920 | 2 | 8 | 4,10 |
| 930 | 2 | 8 | 4,10 |
| 940 | 5 | 1 | 4,10,15,16 |
| 950 | 5 | 1 | 4,10,15,16 |
| 1000 | 2 | 2,13 | 4,10 |
| 1010 | 2 | 1 | 1 |
| 1020 | 2 | 1 | 1 |
| 1030 | 2 | 1 | 1 |
| 1040 | 5 | 1 | 4,10 |
| 1050 | 2 | 7 | 4,12,13 |
| 1100 | 2 | 1 | 4,15,16 |
| 1110 | 2 | 1 | 1 |
| 1120 | 2 | 1 | 4 |
| 1130 | 5 | 1 | 4,10 |
| 1140 | 2 | 1 | 10 |
| 1150 | 2 | 7 | 4,10 |
| 1200 | 2 | 7 | 4,10 |
| 1210 | 2 | 1 | 5,10 |

| | | | |
|---|---|---|---|
| 1220 | 2 | 1 | 5,10 |
| 1230 | 2 | 1 | 5,10 |
| 1250 | 2 | 1 | 1 |
| 1300 | 2 | 1 | 1 |
| 1310 | 2 | 1 | 1 |
| 1320 | 2 | 1 | 1 |
| 1330 | 2 | 1 | 1 |
| 1340 | 2 | 1 | 1 |
| 1350 | 2 | 1 | 1 |
| 1400 | 2 | 1 | 1 |
| 1410 | 5 | 1 | 10 |
| 1420 | 1 | 1 | 10,12,13 |
| 1430 | 2 | 1 | 10 |
| 1440 | 2 | 1 | 1 |
| 1450 | 2 | 1 | 1 |
| 1500 | 2 | 10 | 4,10 |
| 1510 | 2 | 10 | 4,10 |
| 1530 | 2 | 10 | 4,10 |
| 1540 | 2 | 10 | 4,10 |
| 1550 | 2 | 10 | 4 |
| 1600 | 2 | 10 | 4 |
| 1610 | 2 | 10 | 4 |
| 1620 | 2 | 10 | 4 |
| 1630 | 2 | 10 | 4 |
| 1640 | 2 | 10 | 4 |
| 1650 | 2 | 10 | 4 |
| 1700 | 2 | 10 | 4,10 |
| 800 | 2 | 1 | 1 |
| 810 | 2 | 1 | 1 |
| 820 | 2 | 1 | 5,6,15,16 |
| 830 | 2 | 1 | 5,6 |
| 840 | 2 | 1 | 1 |
| 850 | 2 | 5,6 | 7,9 |
| 900 | 1 | 5,6 | 7,15,16 |
| 910 | 1 | 5,6 | 4,7,15,16 |
| 920 | 1 | 5,6 | 4,7,15,16 |
| 930 | 2 | 1 | 1 |
| 940 | 2 | 1 | 1 |
| 950 | 2 | 1 | 1 |
| 1000 | 2 | 1 | 1 |
| 1010 | 2 | 2,3,13 | 4 |
| 1020 | 2 | 1 | 1 |
| 1030 | 2 | 1 | 1 |
| 1040 | 2 | 1 | 1 |
| 1050 | 2 | 1 | 1 |
| 1100 | 2 | 1 | 1 |
| 1110 | 2 | 1 | 1 |
| 1120 | 2 | 5 | 4,15 |
| 1130 | 2 | 1 | 1 |
| 1140 | 2 | 1 | 1 |

| | | | |
|---|---|---|---|
| 1150 | 2 | 1 | 1 |
| 1200 | 2 | 1 | 1 |
| 1210 | 2 | 5,6 | 5,6 |
| 1220 | 2 | 5 | 5,7 |
| 1230 | 2 | 1 | 5,7 |
| 1250 | 2 | 1 | 7 |
| 1300 | 2 | 1 | 7 |
| 1310 | 2 | 5,6 | 11,15,16 |
| 1320 | 2 | 1 | 1 |
| 1330 | 2 | 1 | 1 |
| 1340 | 2 | 1 | 1 |
| 1350 | 2 | 1 | 1 |
| 1400 | 2 | 1 | 1 |
| 1410 | 2 | 1 | 1 |
| 1420 | 2 | 1 | 1 |
| 1430 | 2 | 1 | 1 |
| 1440 | 2 | 1 | 1 |
| 1450 | 2 | 1 | 1 |
| 1500 | 2 | 1 | 1 |
| 1510 | 2 | 1 | 1 |
| 1530 | 2 | 1 | 1 |
| 1540 | 2 | 1 | 1 |
| 1550 | 2 | 1 | 1 |
| 1600 | 2 | 1 | 1 |
| 1610 | 2 | 1 | 1 |
| 1620 | 2 | 1 | 1 |
| 1630 | 2 | 1 | 1 |
| 1640 | 2 | 1 | 1 |
| 1650 | 2 | 1 | 1 |
| 1700 | 2 | 1 | 1 |
| 800 | 2 | 1 | 5,10,15,16 |
| 810 | 2 | 1 | 5,10,15,16 |
| 820 | 2 | 1 | 1 |
| 830 | 2 | 1 | 1 |
| 840 | 2 | 1 | 1 |
| 850 | 2 | 1 | 1 |
| 900 | 2 | 3 | 9 |
| 910 | 2 | 1 | 15,16 |
| 920 | 2 | 1 | 15,16 |
| 930 | 2 | 1 | 1 |
| 940 | 2 | 2,13 | 4,10 |
| 950 | 2 | 2,13 | 4,10 |
| 1000 | 2 | 1 | 1 |
| 1010 | 2 | 1 | 1 |
| 1020 | 2 | 1 | 1 |
| 1030 | 2 | 1 | 1 |
| 1040 | 2 | 1 | 1 |
| 1050 | 2 | 8 | 4,10 |

| | | | |
|---|---|---|---|
| 1100 | 2 | 7 | 4,15,16 |
| 1110 | 1 | 1 | 12,13 |
| 1120 | 2 | 1 | 1 |
| 1130 | 2 | 1 | 1 |
| 1140 | 2 | 1 | 1 |
| 1150 | 2 | 1 | 1 |
| 1200 | 2 | 1 | 1 |
| 1210 | 2 | 1 | 5 |
| 1220 | 2 | 1 | 5,10 |
| 1230 | 2 | 1 | 5,10 |
| 1250 | 2 | 1 | 1 |
| 1300 | 2 | 1 | 1 |
| 1310 | 2 | 1 | 1 |
| 1320 | 2 | 1 | 1 |
| 1330 | 2 | 1 | 1 |
| 1340 | 2 | 1 | 1 |
| 1350 | 2 | 1 | 1 |
| 1400 | 2 | 1 | 1 |
| 1410 | 2 | 1 | 1 |
| 1420 | 2 | 1 | 1 |
| 1430 | 2 | 1 | 1 |
| 1440 | 2 | 1 | 1 |
| 1450 | 7 | | 17 |
| 1500 | 7 | | 17 |
| 1510 | 7 | | 17 |
| 1530 | 7 | | 17 |
| 1540 | 2 | 10 | 4 |
| 1550 | 2 | 10 | 4 |
| 1600 | 2 | 10 | 4 |
| 1610 | 2 | 10 | 4 |
| 1620 | 2 | 1 | 13 |
| 1630 | 1 | 1 | 13 |
| 1640 | 2 | 1 | 10 |
| 1650 | 2 | 1 | 1 |
| 1700 | 2 | 1 | 10 |
| 800 | 1 | 1 | 12 |
| 810 | 1 | 1 | 12 |
| 820 | 2 | 1 | 1 |
| 830 | 2 | 1 | 1 |
| 840 | 2 | 1 | 1 |
| 850 | 2 | 1 | 1 |
| 900 | 2 | 1 | 4 |
| 910 | 2 | 1 | 4 |
| 920 | 2 | 1 | 1 |
| 930 | 2 | 1 | 1 |
| 940 | 2 | 1 | 1 |
| 950 | 2 | 1 | 1 |
| 1000 | 2 | 2,13 | 4,10 |
| 1010 | 2 | 1 | 1 |
| 1020 | 2 | 5 | 15 |

| | | | |
|---|---|---|---|
| 1030 | 2 | 1 | 1 |
| 1040 | 2 | 1 | 1 |
| 1050 | 2 | 1 | 1 |
| 1100 | 2 | 1 | 12,13 |
| 1110 | 2 | 1 | 1 |
| 1120 | 2 | 1 | 1 |
| 1130 | 2 | 1 | 1 |
| 1140 | 2 | 1 | 1 |
| 1150 | 2 | 1 | 1 |
| 1200 | 2 | 10 | 4,10 |
| 1210 | 2 | 10 | 5 |
| 1220 | 2 | 10 | 5,10 |
| 1230 | 2 | 10 | 5,10 |
| 1250 | 2 | 10 | 4 |
| 1300 | 2 | 10 | 4 |
| 1310 | | | 17 |
| 1320 | | | 17 |
| 1330 | | | 17 |
| 1340 | | | 17 |
| 1350 | | | 17 |
| 1400 | | | 17 |
| 1410 | | | 17 |
| 1420 | | | 17 |
| 1430 | | | 17 |
| 1440 | | | 17 |
| 1450 | | | 17 |
| 1500 | | | 17 |
| 1510 | | | 17 |
| 1530 | | | 17 |
| 1540 | | | 17 |
| 1550 | | | 17 |
| 1600 | | | 17 |
| 1610 | | | 17 |
| 1620 | | | 17 |
| 1630 | | | 17 |
| 1640 | | | 17 |
| 1650 | | | 17 |
| 1700 | | | 17 |
| 800 | 2 | 1 | 1 |
| 810 | 2 | 1 | 1 |
| 820 | 2 | 1 | 1 |
| 830 | 2 | 1 | 1 |
| 840 | 2 | 1 | 1 |
| 850 | 2 | 1 | 1 |
| 900 | 2 | 5 | 1 |
| 910 | 2 | 5 | 1 |
| 920 | 2 | 5 | 1,15,16 |
| 930 | 2 | 5,6 | 1,15,16 |
| 940 | 2 | 1 | 1 |
| 950 | 2 | 1 | 1 |

| | | | |
|---|---|---|---|
| 1000 | 2 | 1 | 1 |
| 1010 | 2 | 2,13 | 1 |
| 1020 | 2 | 1 | 1 |
| 1030 | 2 | 5 | 1 |
| 1040 | 2 | 5 | 1 |
| 1050 | 2 | 1 | 1 |
| 1100 | 2 | 1 | 1 |
| 1110 | 2 | 1 | 1 |
| 1120 | 2 | 1 | 1 |
| 1130 | 2 | 1 | 1 |
| 1140 | 2 | 2,3,5 | 1 |
| 1150 | 2 | 2,3,5 | 1 |
| 1200 | 2 | 1 | 1 |
| 1210 | 2 | 3 | 1 |
| 1220 | 2 | 1 | 1 |
| 1230 | 2 | 1 | 1 |
| 1250 | 2 | 1 | 1 |
| 1300 | 2 | 1 | 1 |
| 1310 | 2 | 1 | 1 |
| 1320 | 2 | 1 | 1 |
| 1330 | 2 | 1 | 1 |
| 1340 | 2 | 1 | 1 |
| 1350 | 2 | 1 | 1 |
| 1400 | 2 | 3,5,6 | 1 |
| 1410 | 2 | 7,8 | 1 |
| 1420 | 2 | 1 | 1 |
| 1430 | 2 | 1 | 1 |
| 1440 | 2 | 1 | 1 |
| 1450 | 2 | 1 | 1 |
| 1500 | 2 | 1 | 1 |
| 1510 | 2 | 1 | 1 |
| 1530 | 2 | 1 | 1 |
| 1540 | 2 | 1 | 1 |
| 1550 | 2 | 1 | 1 |
| 1600 | 2 | 1 | 1 |
| 1610 | 2 | 1 | 1 |
| 1620 | 2 | 1 | 1 |
| 1630 | 2 | 1 | 1 |
| 1640 | 2 | 1 | 1 |
| 1650 | 2 | 1 | 1 |
| 1700 | 2 | 1 | 1 |
