# Supplementary material for: A comparative study of patients’ activities and interactions in a stroke unit before and after reconstruction—The significance of the built environment
Source: PLoS One. 2017 Jul 20;12(7):e0177477. doi: 10.1371/journal.pone.0177477 (PMC5519004; doi:10.1371/journal.pone.0177477)
Supplement: S3 Fig — (PDF) [file pone.0177477.s003.pdf]

TIME	Location	People	Activity	Location	People	Activity	Location	People
800	2	5,6	1	2	1	1	2	1
810	2	5	1	1	8	12,15	2	1
820	2	3	4,12	2	8	7,15,16	2	1
830	5	1	4,7	5	1	4,7	2	3
840	2	3	4,5,7	2	1	7	5	1
850	2	1	7	5	1	4,5,7	5	1
900	2	1	7	2	1	7	5	1
910	2	1	7	2	1	7	5	1
920	2	1	7	2	1	7	5	1
930	2	1	4,6	2	1	1	2	1
940	2	1	1,7	2	1	1	2	1
950	2	1	1,7	2	1	1	2	1
1000	2	1	7	2	1	1	2	1
1010	2	1	1	2	1	1	2	1
1020	2	1	4,7	2	1	1	2	1
1030	2	5	4,7	2	1	1	2	1
1040	2	1	1	2	1	1	2	1
1050	2	1	1	2	1	1	2	1
1100	2	1	1	2	1	4	2	1
1110	2	1	1	2	13	4,6	2	1
1120	1	3,5	13	2	13	4,6	2	1
1130	2	1	1	2	1	1	2	1
1140								
1150	2	8	4,7	5	1	5,7	5	1
1200	2	1	4,7	5	1	5,7	5	1
1210	3	4	13	5	1	5,7	5	1
1220	5	1	5	5	1	5	5	5
1230	5	1	5,7	5	1	4,5,7	5	1
1240	2	1	4,7	2	1	7	2	1
1250	2	1	1	2	5	1	2	10
1300	2	1	4,7	2	1	1	2	10
1310	2	1	4,7	2	1	1	2	10
1320	2	1	7	2	1	1	2	1
1330	2	1	7	2	1	1	2	1
1340	2	1	4,7	2	1	1	2	1
1350	2	1	7	2	1	1	2	1
1400	2	1	1	2	1	1	2	7
1410	2	1	1	2	1	1	2	1
1420	2	1	7	2	1	1	2	1
1430	2	1	7	2	1	1	2	1
1440	2	1	1	2	1	1	2	1
1450	2	1	1	2	7	13	2	1
1500	2	1	1	2	5	7	2	1
1510	2	1	1	2	1	6	2	1
1520	2	1	1	3	10	4,13	2	1
1530	2	1	5,7	2	1	5,7	2	1
1540	2	1	5,7	2	1	5,7	2	1
1550	2	1	7	2	1	7	2	1
1600	2	1	7	5	1	5,7	2	1

1610	2	3	7	2	1	5,7	2	1
1620	2	1	7	3	7	13	2	1
1630	2	1	7	3	7	13	2	1
1640	2	1	7	2	1	7	2	1
1650	2	5	5,7	1	1	17	2	1
1700	2	1	7	1	1	17	2	1
800	2	1	1	2	1	1	2	1
810	2	1	1	2	1	1	2	1
820	2	5,6	4,6	2	1	1	2	1
830	1	5,6	17	2	1	5,6	2	1
840	2	5,6	4,8				2	1
850	2	3,4,5	4,7	2	1	1	5	1
900	2	1	5,6	2	1	5,6	5	1
910	5	1	5,7	2	1	1	2	1
920	2	1	7	2	1	1	2	1
930	2	1	7	2	1	1	2	1
940	2	5	5,7	2	1	1	2	1
950	2	1	4,7	2	1	1	2	1
1000	2	5	7	2	1	1	2	8
1010	2	1	1,7	2	7	7,15,16	2	1
1020	2	1	1	2	1	7	2	7
1030	2	1	1,4	2	1	1	2	2
1040	2	3	1	5	1	7	2	1
1050	2	1	1	5	1	7	2	1
1100	2	1	1	5	1	7	7	5
1110	2	1	1	5	1	7	7	5
1120	2	7	4,6,15,16	5	1	7	3	1
1130	2	1	1	2	1	7	2	1
1140	2	1	1	2	1	1,7	2	1
1150	2	1	1	2	1	7	9	5
1200	2	1	1	2	1	1	9	5
1210	2	1	1	5	1	5,7	9	5
1220	2	1	5,6	5	1	5,7	9	5
1230								
1240	2	1	1	5	1	5,7	2	1
1250	1	3,4	7	5	1	4,7	2	1
1300	2	5	5,6				2	1
1310	2	1	1	2	8	4,7	2	1
1320	2	1	1	2	8	4,6,15,16	2	1
1330	2	1	1	2	1	1	2	1
1340	2	1	1	2	1	1	2	1
1350	2	3	1,4	2	1	1	2	1
1400	2	1	1	2	1	1	2	1
1410	2	1	1	2	1	1	2	1
1420	2	1	1	2	1	1	2	1
1430	2	1	1	2	1	1	2	1
1440	2	5,6	4,6,9	2	1	1	2	1
1450	2	1	1	2	1	1	2	1
1500	2	1	1	2	1	1	2	1
1510	2	1	1	2	1	1	3	7

1520	2	1	1	2	1	1	3	7
1530	2	1	1	2	1	1	3	7
1540	2	1	1	2	1	1	2	10
1550	2	1	1	2	1	1	2	10
1600	2	1	1	2	1	1	2	10
1610	2	4	1	2	1	1	2	10
1620	2	1	1	2	1	1	2	5,10
1630	2	1	1	2	1	1	2	10
1640	2	1	1	2	1	1	2	10
1650	1	5	17	2	1	1	2	10
1700	2	5,6	7	2	1	1	2	10
800	2	5	1,4	2	1	1	2	5
810	2	5	6	2	1	1	2	5
820	5	1	5,7	5	1	5,7	2	1
830	5	1	5,7	5	1	7	2	1
840	5	1	5,7	5	1	5,7	2	1
850	3	1	11,13	5	1	7	2	3,10
900	2	1	7	2	1	7	6	2,10
910	2	1	7	2	1	7	6	2,10
920	2	1	1	2	1	7	2	1
930	2	1	1	2	1	7	2	1
940	2	1	1	2	1	7	2	1
950	2	1	1	3	7	4,13	2	10
1000	2	1	1	4	7	11,13	2	1
1010	2	7	11,12	4	7	11,12,13	2	10
1020	2	1	1	4	7	12	2	1
1030	2	1	1	2	1	1	2	10
1040	2	10	4,6	2	1	1	9	10
1050	2	10	4,6	2	1	1	9	10
1100	2	10	7	2	1	1	9	10
1110	2	10	7	2	1	1	9	10
1120								
1130	2	1	7	2	8	4,6	2	1
1140	2	10	4,7	2	1	1,4	2	1
1150	2	1	7	2	1	1	2	5
1200	6	3,10,13	4,7	2	1	1	2	1
1210	6	3,10,13	7	2	1	7	2	1
1220	6	3,10,13	4,7	2	1	1	2	1
1230	3	10	13	2	1	1	2	1
1240	5	1	5,7	2	1	1	2	1
1250	3	1	13	2	1	1	2	1
1300	2	1	1	2	1	1	2	1
1310	2	1	1	2	1	1	2	1
1320	2	1	1	2	1	1	2	1
1330	1	1	17	2	1	1	2	1
1340	2	1	1	2	1	1	2	1
1350	1	1	13	1	1	1	2	1
1400	2	1	7	2	1	1	2	1
1410	3	1	13	1	1	1	1	1
1420	2	1	7	2	1	1	2	1

1430	2	1	7	2	1	1	2	1
1440	2	1	7	2	1	1	2	1
1450	2	1	1	2	1	1	2	1
1500	2	1	1	2	1	1	2	1
1510	5	1	5,7	5	1	5,7	2	1
1520	2	1	1	2	1	1	2	1
1530	2	1	1	2	1	1	2	1
1540	2	1	1	2	1	1	2	1
1550	2	1	1	2	1	1	2	1
1600	2	1	1	2	1	1	2	1
1610	2	1	1	2	1	1	2	1
1620	2	1	1,3	2	5	1,4	2	1
1630	3	1	13	2	1	1	2	1
1640	5	1	5,7	5	1	5,7	2	1
1650	5	1	4,5,7	5	1	5,7	2	1
1700	2	1	7	5	1	7	2	1

Activity	Location	People	Activity	Location	People	Activity
1	2	1	1	2	1	1
1	2	1	1	2	1	6
1	2	3,5	7	2	1	7
	2	1	1	2	1	7
5,7	2	1	7	2	1	7
5,7	5	1	5,7	5	1	7
7	5	1	5,7	2	1	7
7	5	1	5,7	2	1	7
5,7	2	1	1	2	1	12
1	2	1	1	2	1	1
1	3	1	13	2	1	1
1	3	1	13	2	1	1
1	2	1	1	2	1	1
1	2	1	4,7	2	1	1
1	2	1	1	2	1	1
1	2	1	1	2	1	1
1	2	1	1	2	1	1
1	2	1	1	2	1	1
1	2	1	1	2	1	1
1	2	1	1	2	1	1
1	2	1	1	2	1	1
1	2	1	1	2	1	1
5,7	2	1	1	5	1	5,7
5,7	2	1	1	5	1	5,7
7	2	1	1	5	1	5,7
5	5	1	5	2	10	4,5,13
7	5	1	7	2	10	5,6
1	2	1	1	2	1	7
1	2	1	1	2	1	7
6	2	1	1	2	10	7
4,6	2	1	1	2	1	1
1	3	1	7	2	1	1
1	2	1	1	2	1	1
1	2	1	1	2	1	1
1	3	7	13	2	1	4,7
11,12	3	5	7	2	1	6
1	2	1	1	2	10	1
1	2	1	1	2	1	4,7
1	2	1	1	2	1	4,7
1	2	1	1	2	10	4,5,7
1	2	1	1	2	10	1
1	2	5	1	2	1	1
1	2	1	1	2	1	1
1	2	1	1	2	1	1
1	2	1	1	2	1	1
1	2	1	1	3	7	13
1	2	1	1	2	1	1
1	2	1	1	2	10	7

1	2	1	1	2	10	7
1	2	1	1	2	1	1
1	2	1	1	2	1	1
1	2	1	1	2	1	1
1	2	1	1	2	1	1
1	2	1	1	2	10	1,4
1	2	1	6	2	1	1
1	2	1	5,6	2	5	1,4
1	2	3	5,6	2	5	7
1	1	1	17	2	5	7
5,6	2	1	1	5	5	5,7
5	2	1	1	2	1	1
5,7	2	1	6	5	5	7
4,7	2	1	6	5	1	5,7
4,7	2	1	1	5	1	5,7
4,7	2	1	1	5	1	7
6	2	1	5,6	5	1	7
1	2	1	1	5	1	7
4,7	2	1	1	5	1	7
4,6	2	1	1	2	1	7
1,4	2	1	1	3	1	7
4,6	2	1	1	3	1	7
1	2	1	1	3	1	7
1	2	1	1	3	1	7
17	2	1	1	2	1	7
17	2	1	1	2	1	7
13	2	1	1	2	3	4,6
4	2	9	1	2	1	1
4,7	2	1	6	2	1	1
17	2	1	1	2	1	1
17	2	1	1	2	1	1
17	2	1	1	2	3	7
17	2	1	1	5	5	5,7
1	2	1	5,6	2	1	7
5,6	2	1	1	5	1	5,7
1	2	1	1	5	1	7
1	2	1	1	2	1	7
1	2	1	1	2	1	7
1	2	1	1	3	1	7
1	2	1	1	3	1	7
1	2	1	1	3	1	7
1	2	1	1	3	1	7
1	2	1	1	2	1	1
1	2	1	1	2	1	1
1	2	1	1	2	1	1
4,7	2	1	1	2	1	1
1	2	1	1	2	1	1
1	2	1	1	2	1	1
4,13	2	1	1	2	5	6

13	2	1	1	2	1	1
4,12	2	1	1	2	1	1
4,7	5	10	4,5,7	2	1	1
4,7	2	10	4,6	2	1	1
4,7	2	10	4,6	2	1	1
4,7	2	10	4,6	2	1	1
4,5,7	5	10	5,7	2	1	1
5,7	5	1	5	2	1	1
1	2	1	1	2	10	4,6
1,4	2	1	1	2	10	4,6
1,4	2	1	1	2	10	4,6
1	2	1	1	2	1	1
6	2	1	1	2	1	1
5,7	2	1	1	2	1	5,7
7	2	1	1	2	1	7
5,7	2	1	1	2	1	7
4,7	2	5	4,9	2	1	7
4,7	2	1	1	2	1	7
4,7	2	1	1	2	1	7
7	2	1	1	2	1	1
7	2	1	1	2	1	1
1	2	1	6	1	1	13
1	2	1	1	2	1	1
1	2	1	1	2	1	1
4,7	2	3	1,4	2	1	1
1	2	1	1	2	1	7
7	1	10	7	2	1	1
17	2	3,10	1,4	2	1	1
17	2	10	6	2	1	1
17	2	10	4,9	1	1	17
17	2	10	1,4	2	1	7
7,15	2	1	6	2	1	7
7	2	1	6	2	1	7
12	2	8	4,7	2	1	7
7	2	8	4,7	2	1	7
7	2	8	4,7	2	1	7
1	2	1	5,7	2	1	1
1	2	1	5,7	2	5	5,7
1	2	1	5,7	2	1	5,7
7	2	1	1	2	1	5,7
7	2	1	7	2	1	1
7	2	1	7	2	1	1
7	2	1	7	2	1	1
1	2	1	1	2	1	1
1	2	1	1	2	1	1
1	2	1	1	2	1	1
1	2	1	1	2	1	1
1	1	1	1	1	1	1
1	2	1	7	2	1	7

	1	2	1		7	2	1		7
	1	2	1		7	2	1		7
	1	2	1		1	4	7	4,11,13	
	1	2	1		1	4	7	5,13	
	1	2	1	5,7		2	1	5,7	
	1	2	1		7	2	1		7
	1	2	1		1	1	1		17
	1	2	1		7	2	1		7
	1	2	1		7	2	1		7
	1	2	1		7	2	1		7
	1	2	1		7	2	1		7
	1	2	1		7	2	1		7
	1	2	1		7	2	1		7
	1	2	1	5,7		2	1	5,7	
5,7		2	1	5,7		2	1	5,7	
	7	2	1	5,7		2	1	5,7	
